# Online evaluation method of coal mine comprehensive level based on FCE

**DOI:** 10.1371/journal.pone.0256026

**Published:** 2021-08-16

**Authors:** Ling Shen, Guoxun Jing, Qiang Zeng

**Affiliations:** 1 School of Safety Science and Engineering, Henan Polytechnic University, Henan Jiaozuo, China; 2 Anyang Institute of Technology, Henan Anyang, China; 3 School of Business Administration, Henan Polytechnic University, Henan Jiaozuo, China; Universita degli Studi del Molise, ITALY

## Abstract

An online evaluation method of coal mine comprehensive level based on Fuzzy Comprehensive Evaluation method (FCE) is proposed. Firstly, following the principles of fairness, systematicness and hierarchy, taking research and development, production, sales, finance, safety and management as the first level indicators, a set of multi-level evaluation indicator system of coal mine comprehensive level combining objective and subjective evaluation indicators is established. Secondly, according to the characteristics of the indicator system, the specific process of FCE of coal mine comprehensive level is given. Then, taking SQL Server as the database management system and C#.NET as the development language, a set of B/S structure online evaluation system of coal mine comprehensive level based on FCE is designed and developed. Finally, the proposed method is applied to Coal group PM for test. The application shows that the method proposed can provide an efficient and convenient online evaluation platform to evaluate the comprehensive level of coal mines for the Coal group, and the horizontal and longitudinal comparison of the evaluation results can urge the coal mines to maintain their advantages and avoid their disadvantages, which is of some significance for improving the overall competitiveness of the Coal group.

## 1. Introduction

Coal mines are important economic cells that provide coal resources for a country. As a Coal group, it is of some significance to evaluate the comprehensive level of the coal mines under its jurisdiction and promote improvement through evaluation for improving the overall competitiveness of the coal mines and even the Coal group.

Current research about coal mine evaluation mainly includes safety evaluation [1–3], risk evaluation [4–7], ecological environment evaluation [8, 9], system evaluation [10], science and technology evaluation [11], etc. These belong to professional evaluations from a certain point of view, which may fall into the one-sided. As a production-oriented enterprise, we think that comprehensive evaluation including research and development, production, sales, finance, management, etc. has more guiding significance.

The comprehensive level evaluation of coal mines belongs to multi-criteria evaluation. Common methods of multi-criteria evaluation include Delphi Method [12, 13], Analytic hierarchy process method (AHP) [14–16], Weight summation method (WSM), Weight product method (WPM), Entropy method [17, 18], Factor analysis method (FA) [19], TOPSIS method [20–22], Artificial neural network method (ANN) [23, 24], Multiple regression analysis method (MRA) [25, 26], Fuzzy comprehensive evaluation method (FCE) [27–29], etc. Among them, Delphi method and AHP method are suitable for evaluation of the subjective evaluation indicators. WSM method, WPM method, Entropy method, FA method and TOPSIS method are suitable for the evaluation of the objective evaluation indicators. ANN method, MRA method and FCE method are all suitable for the evaluation of the subjective and objective indicators. However, ANN method and MRA method are not suitable for the evaluation of multi-level indicators. FCE method is suitable for the evaluation of multi-level indicators. Through FCE method not only can the overall evaluation result be obtained, but also can the evaluation results of each indicator be obtained, which makes it easy to find out the disadvantages and propose corresponding improvement measures.

In the aspect of evaluation operation, the informationalized method of coal mine evaluation needs to be improved urgently. With the advent of the information age, manual or stand-alone evaluation has more and more exposed its shortcomings. For example, the evaluation has a certain space limitation, evaluation and calculation efficiency is low, it is difficult to make evaluation results be shared and compared.

Based on above analysis, an online evaluation method of coal mine comprehensive level based on FCE is put forward. Following the principles of fairness, systematicness and hierarchy, a set of multi-level evaluation indicator system for coal mine comprehensive level is established. The specific process of FCE for coal mine comprehensive level is given. Taking SQL Server as the database management system and C#.NET as the development language, a set of online evaluation system for comprehensive level of coal mines is designed and developed. The proposed method is applied to Coal group PM for test.

## 2. Establishing of evaluation indicator system

Following the principles of fairness, systematicness and hierarchy, through literature search and investigation of Coal group PM, a set of multi-level evaluation indicator system for coal mine comprehensive level is established, which takes research and development, production, sales, finance, safety and management as the first-level indicators, as shown in Table 1. The specific process is as follows. Firstly, a draft indicator system is constructed through literature search. Secondly an expert group consisting of 18 experts coming from Coal group PM and the coal mines of Coal group PM is established. Thirdly, the rationality of the selected indicators is discussed through expert meetings. Finally, the weight of each indicator is determined one by one through expert meetings.

**Table 1:** Multi-level evaluation indicator system of coal mine comprehensive level.

Level 1	Weight	Level 2	Weight	Level 3	Weight	Explanation
Research and development	0.15	Has a R&D team	0.2			Objective indicator, hoping-have
		Has any R&D plan	0.15			Objective indicator, hoping-have
		Research funding per million tons	0.15			Objective indicator, hoping-large, yuan/million tons
		Number of research awards per million tons	0.2			Objective indicator, hoping-large, item/million tons
		Number of invention patents per million tons	0.2			Objective indicator, hoping-large, item/million tons
		Number of utility model patents per million tons	0.1			Objective indicator, hoping-large, item/million tons
Production	0.2	Production cost per ton	0.3			Objective indicator, hoping-small, yuan/ton
		Profit per ton	0.3			Objective indicator, hoping-small, yuan/ton
		Daily output per employee	0.2			Objective indicator, hoping-large, ton/person/day
		Energy consumption per ton	0.2			Objective indicator, hoping-small, degree/ton
Sales	0.15	Energy consumption per ton	0.2			Subjective indicator
		Integrity degree of sales team	0.25			Subjective indicator
		Logistics accessibility	0.25			Subjective indicator
		Profit margin on sales	0.3			Objective indicator, hoping-large
Finance	0.15	Return rate on total assets	0.5			Objective indicator, hoping-target, target = 0.5
		Asset liability ratio	0.5			Objective indicator, hoping-have
Safety	0.2	Gas accident	0.25	Has gas accident prevention measures	0.3	Objective indicator, hoping-have
				Has emergency measures for gas accident	0.3	Objective indicator, hoping-have
				Has gas accidents	0.4	Objective indicator, hoping-no
		Roof caving	0.25	Has roof caving prevention measures	0.3	Objective indicator, hoping-have
				Has emergency measures for roof caving	0.3	Objective indicator, hoping-have
				Has roof caving accidents	0.4	Objective indicator, hoping-no
		Coal outburst	0.25	Has coal outburst prevention measures	0.3	Objective indicator, hoping-have
				Has emergency measures for coal outburst	0.3	Objective indicator, hoping-have
				Has coal outburst accidents	0.4	Objective indicator, hoping-no
		Water accident	0.25	Has water accident prevention measures	0.3	Objective indicator, hoping-have
				Has emergency measures for water accident	0.3	Objective indicator, hoping-have
				Has water accident accidents	0.4	Objective indicator, hoping-no
Management	0.15	Managers	0.35	Leading ability	0.2	Subjective indicator
				Organization ability	0.2	Subjective indicator
				Decision ability	0.2	Subjective indicator
				Planning ability	0.2	Subjective indicator
				Coordinating ability	0.2	Subjective indicator
		Employees	0.3	Basic quality	0.3	Subjective indicator
				Belonging sense degree	0.3	Subjective indicator
				Executive force	0.4	Subjective indicator
		Culture	0.35	Learning culture	0.3	Subjective indicator
				Safety culture	0.4	Subjective indicator
				Environmental culture	0.3	Subjective indicator

## 3. Process design of FCE

It can be seen from Table 1 that the evaluation indicator system is a multi-level evaluation indicator system including objective evaluation indicators and subjective evaluation indicators. For this kind of evaluation indicator system, FCE method is suitable for evaluation. The quantitative indicators (hoping-large, hoping-target, hoping-small) and have-no indicators (hoping-have, hoping-no) belong to objective evaluation indicators. The qualitative indicators belong to the subjective indicators. For the objective evaluation indicators, no matter which expert evaluates it, the evaluation result is the same, so the same indicator only needs to be evaluated once. For the subjective evaluation indicators, it depends on the subjective judgment of the experts to give their grades, so the same indicator usually needs to be evaluated for more than once by different experts. Based on this, the specific process of FCE designed in this paper is as follows.

1. Evaluation grading. Select grade A, B, C, D, E to evaluate the coal mines.
2. Evaluation of final-level indicators. For the final-level subjective evaluation indicators, invite several experts to give grade A, B, C, D or E for each indicator. For the final-level objective evaluation indicators (hoping-large, hoping-target, hoping-small, hoping-have, hoping-no), invite one or more experts to give numeric value for each indicator according to their own expertise. Among them, for the hoping-have or hoping-no indicators, 1 or 0 should be given, 1 represents “have” and 0 represents “no”. Different from the subjective evaluation indicators, the same objective evaluation indicator is only evaluated once.
3. Membership vector determination of final-level indicators.

1. For the final-level subjective evaluation indicator x, apply statistical method to determine its membership vector *U*(*x*). The specific method is as follows. Count the number of grade *A*, *B*, *C*, *D*, *E* respectively, and assign them to *n*_*A*_, *n*_*B*_, *nc*, *n*_*D*_, *n*_*E*_. Let *n* = *n*_*A*_+*n*_*B*_+*nc*+*n*_*D*_+*n*_*E*_. Get the membership vector *U*(*x*) according to Formula (1).
$$U(x)=[\begin{array}{ccccc} u_{A}(x) & u_{B}(x) & u_{C}(x) & u_{D}(x) & u_{E}(x) \end{array}]=[\begin{array}{ccccc} \frac{n_{A}}{n} & \frac{n_{B}}{n} & \frac{n_{C}}{n} & \frac{n_{D}}{n} & \frac{n_{E}}{n} \end{array}]$$ (1)
2. For the final-level objective evaluation indicator x, determine its membership vector U(x) according to its characteristics (hoping-large, hoping-target, hoping-small, hoping-have, hoping-no) by appropriate methods.

**Fig 1:**
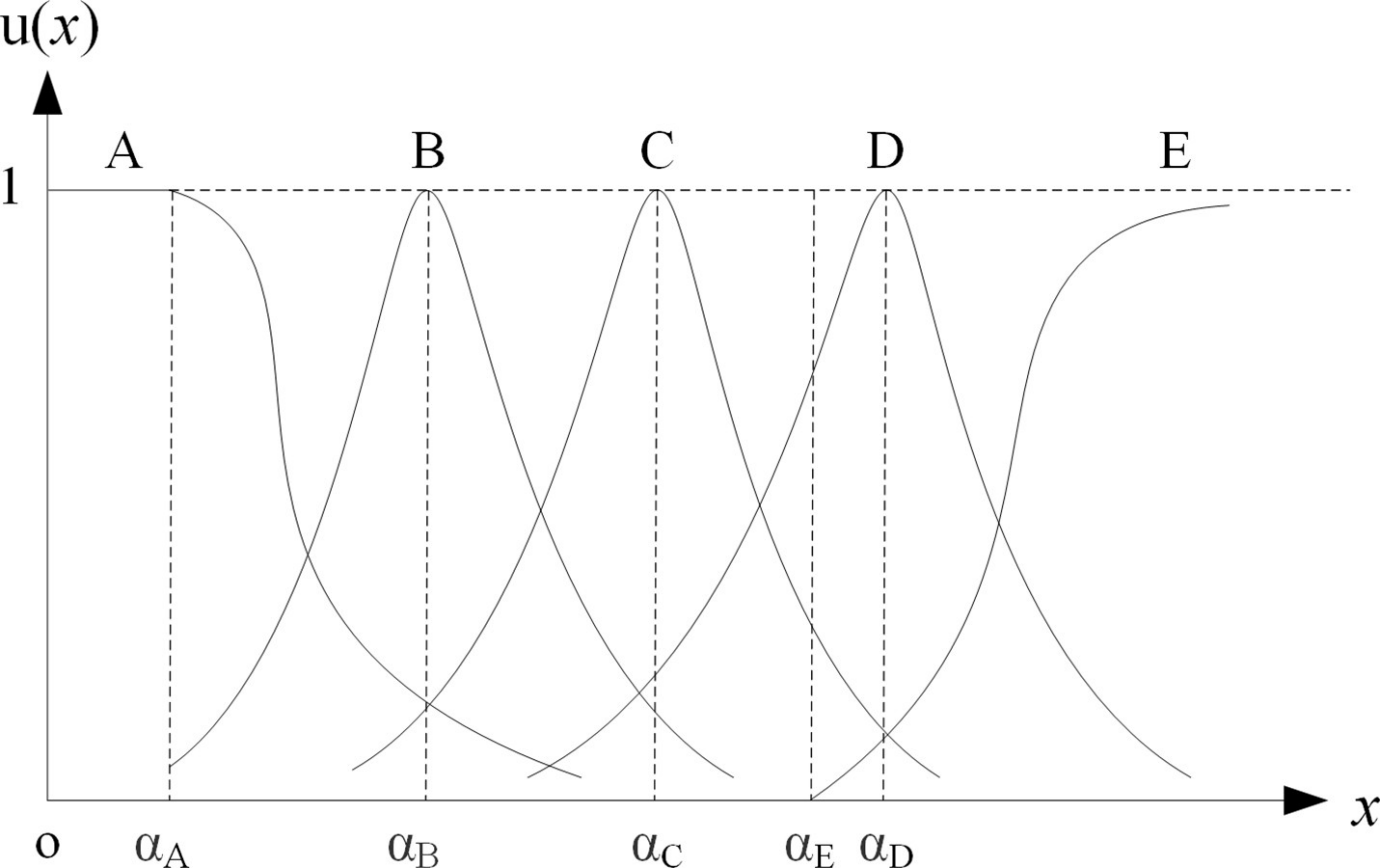
Membership vector of final-level hoping-small indicator.

**Fig 2:**
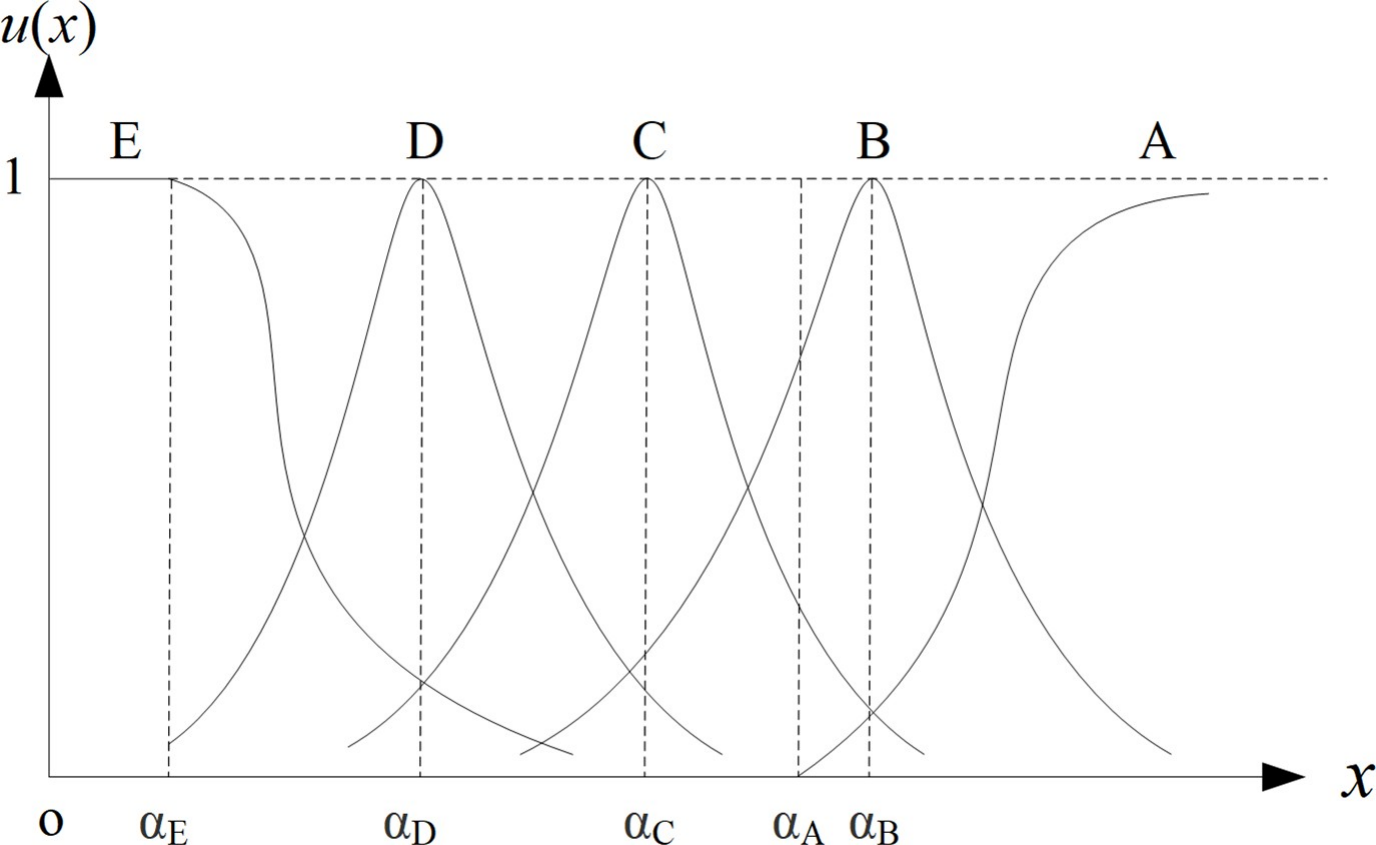
Membership vector of final-level hoping-large indicator.

① Membership vector determination of final-level hoping-small indicators. For the indicator *x*, apply Cauchy-type membership function to determine its membership vector, as shown in Fig 1. Specifically, when *x*≤*α*_*A*_, let *U*(*x*) = [1 0 0 0 0]; when *α*_*A*_ <*x*≤*α*_*E*_, firstly determine *U*^0^(*x*) by Formula (2), then let $u_{A}{}^{0}(x)=\frac{1}{{\left(1+\alpha_{1}(x-\alpha_{A})\right)}^{\beta }}$, $u_{B}{}^{0}(x)=\frac{1}{{\left(1+\alpha_{2}(x-\alpha_{B})\right)}^{\beta }}$, $u_{C}{}^{0}(x)=\frac{1}{{\left(1+\alpha_{3}(x-\alpha_{C})\right)}^{\beta }}$, $u_{D}{}^{0}(x)=\frac{1}{{\left(1+\alpha_{4}(x-\alpha_{D})\right)}^{\beta }}$, $u_{E}{}^{0}(x)=0$, finally normalize it to get *U*(*x*) according to Formula (3); when *x*>*α*_*E*,_ firstly determine *U*^0^(*x*) by Formula (4), then normalize it to get *U*(*x*) according to Formula (3). Among them, *α*_1_, *α*_2_, *α*_3_, *α*_4_, *α*_5_ are adjustment coefficients, whose values can be calculated by substituting the indicator values (*Ah*, *Bh*, *Ch*, *Dh*, *Eh*) with that corresponds to membership degree of 0.5 into the membership formula. Usually let *β* = 2.
② Membership vector determination of final-level hoping-target indicators. For the indicator *x*, suppose the target is m, let *x*’ = |*x*-*m*|. The new indicator *x*’ is a hoping-small indicator. Determine the membership vector *U*(*x*’) of indicator *x*’ by the method described in ①. Finally, let *U*(*x*) = *U*(*x*’).
③ Membership vector determination of final-level hoping-large indicators. For the indicator x, apply Cauchy-type membership function to determine its membership vector, as shown in Fig 2. Specifically, when *x*≤*α*_*E*_, let *U*(*x*) = [1 0 0 0 0]; when *α*_*E*_ <*x*≤*α*_*A*_, firstly determine *U*^0^(*x*) by Formula (5), then normalize it to get *U*(*x*) according to Formula (3); when *x*>*α*_*E*_, firstly determine *U*^0^(*x*) by Formula (6), then normalize it to get *U*(*x*) according to Formula (3).
④ Membership vector determination of final-level hoping-have indicators. For the indicator x, apply grade exchange method to determine its membership vector. Let 1 represent “have” and 0 represent “no”. When *x*≤1, let *U*(*x*) = [1 0 0 0 0]; when *x* = 0, let *U*(*x*) = [0 0 0 0 1].
⑤ Membership vector determination of final-level hoping-no indicators. For the indicator *x*, apply grade exchange method to determine its membership vector. Let 1 represent “have” and 0 represent “no”. When *x* = 1, let *U*(*x*) = [0 0 0 0 1]; when *x* = 1, let *U*(*x*) = [1 0 0 0 0].

$$U^{0}(x)=[\begin{array}{ccccc} \frac{1}{{\left(1+\alpha_{1}(x-\alpha_{A})\right)}^{\beta }} & \frac{1}{{\left(1+\alpha_{2}(x-\alpha_{B})\right)}^{\beta }} & \frac{1}{{\left(1+\alpha_{3}(x-\alpha_{C})\right)}^{\beta }} & \frac{1}{{\left(1+\alpha_{4}(x-\alpha_{D})\right)}^{\beta }} & 0 \end{array}]$$ (2)

$$U(x)=[\begin{array}{ccccc} \frac{u_{A}{}^{0}(x)}{U^{0}} & \frac{u_{B}{}^{0}(x)}{U^{0}} & \frac{u_{C}{}^{0}(x)}{U^{0}} & \frac{u_{D}{}^{0}(x)}{U^{0}} & \frac{u_{E}{}^{0}(x)}{U^{0}} \end{array}]$$ (3)

Among Formula (3), $U^{0}=u_{A}{}^{0}(x)+u_{B}{}^{0}(x)+u_{C}{}^{0}(x)+u_{D}{}^{0}(x)+u_{E}{}^{0}(x)$。

$$U^{0}(x)=[\begin{array}{ccccc} \frac{1}{{\left(1+\alpha_{1}(x-\alpha_{A})\right)}^{\beta }} & \frac{1}{{\left(1+\alpha_{2}(x-\alpha_{B})\right)}^{\beta }} & \frac{1}{{\left(1+\alpha_{3}(x-\alpha_{C})\right)}^{\beta }} & \frac{1}{{\left(1+\alpha_{4}(x-\alpha_{D})\right)}^{\beta }} & \frac{1}{{\left(1+\alpha_{5}(x-\alpha_{E})\right)}^{-\beta }} \end{array}]$$ (4)

$$U^{0}(x)=[\begin{array}{ccccc} 0 & \frac{1}{{\left(1+\alpha_{2}(x-a_{B})\right)}^{\beta }} & \frac{1}{{\left(1+\alpha_{3}(x-a_{C})\right)}^{\beta }} & \frac{1}{{\left(1+\alpha_{4}(x-a_{D})\right)}^{\beta }} & \frac{1}{{\left(1+\alpha_{5}(x-a_{E})\right)}^{\beta }} \end{array}]$$ (5)

$$U^{0}(x)=[\begin{array}{ccccc} \frac{1}{{\left(1+\alpha_{1}(x-a_{A})\right)}^{-\beta }} & \frac{1}{{\left(1+\alpha_{2}(x-a_{B})\right)}^{\beta }} & \frac{1}{{\left(1+\alpha_{3}(x-a_{C})\right)}^{\beta }} & \frac{1}{{\left(1+\alpha_{4}(x-a_{D})\right)}^{\beta }} & \frac{1}{{\left(1+\alpha_{5}(x-a_{E})\right)}^{\beta }} \end{array}]$$ (6)

$$\begin{array}{l} U(x)=[\begin{array}{ccccc} u_{A}(x) & u_{B}(x) & u_{C}(x) & u_{D}(x) & u_{E}(x) \end{array}]\\ =[\begin{array}{ccccc} w_{1} & w_{2} & \cdots & w_{k-1} & w_{k} \end{array}]*\left[\begin{array}{ccccc} u_{A}(x_{1}) & u_{B}(x_{1}) & u_{C}(x_{1}) & u_{D}(x_{1}) & u_{E}(x_{1})\\ u_{A}(x_{2}) & u_{B}(x_{2}) & u_{C}(x_{2}) & u_{D}(x_{2}) & u_{E}(x_{2})\\ \cdots & \cdots & \cdots & \cdots & \cdots \\ u_{A}(x_{k-1}) & u_{B}(x_{k-1}) & u_{C}(x_{k-1}) & u_{D}(x_{k-1}) & u_{E}(x_{k-1})\\ u_{A}(x_{k}) & u_{B}(x_{k}) & u_{C}(x_{k}) & u_{D}(x_{k}) & u_{E}(x_{k}) \end{array}\right] \end{array}$$ (7)

$$u_{A}(x)=\sum_{i=1}^{k}w_{i}u_{A}(x_{i})$$ (8)

1. Membership vector determination of non-final-level indicators. For the non-final-level indicators, apply the weighted average fuzzy operator to determine the membership vector of each indicator from low level to high level. For the non-finial-level indicator *x*, suppose it has *k* sub-indicators which are *x*_1_, *x*_2_, …, *x*_*k*-1_, *x*_*k*_, with weights of *w*_1_, *w*_2_, …, *w*_*k*-1_, *w*_*k*_. Determine its membership vector *U*(*x*) according to Formula (7). Among them, take *u*_*A*_(*x*) for an example, its calculation formula is shown in Formula (8).
2. Membership determination of evaluated coal mine. For the evaluated coal mine, apply the weighted average fuzzy operator to determine its membership vector U according to the first-level indicators. The determination method is the same as that of non-final-level indicators.
3. Grade determination of each indicator and the evaluated coal mine. After membership vector determination of each indicator and the evaluated coal mine, give the grade of them respectively according to their maximum membership degrees. If number of the maximum membership degree are larger than one, take more than one grades. For example, if the membership degrees of both grade A and grade B of an indicator is equal to the maximum membership value of 0.3, then the evaluation grade of the indicator is A or B, denoted as AB.
4. Score determination of each indicator and the evaluated coal mine. In order to reflect the advantages and disadvantages of each indicator and the evaluated coal mine more intuitively, apply the weighted average method to calculate their scores according to 5-point system. Taking the indicator x as an example, its calculation formula is shown in Formula (9).

$$s(x)=5u_{A}(x)+4u_{B}(x)+3u_{C}(x)+2u_{D}(x)+u_{E}(x)$$ (9)

## 4. Design of online evaluation system

It can be seen that the calculation amount of the above evaluation method is large. Manual or stand-alone evaluation method is difficult to meet needs of the Coal group’s evaluation and comparison of the comprehensive level of coal mines under its jurisdiction. In order to improve the evaluation efficiency, ensure the accuracy of calculation results, ensure the sharing of evaluation results and realize the comparison of evaluation results, taking SQL Server as database management system and C#.NET as the development language, a set of online evaluation system for coal mine comprehensive level based on FCE is designed and developed.

### 4.1 Function planning

**Fig 3:**
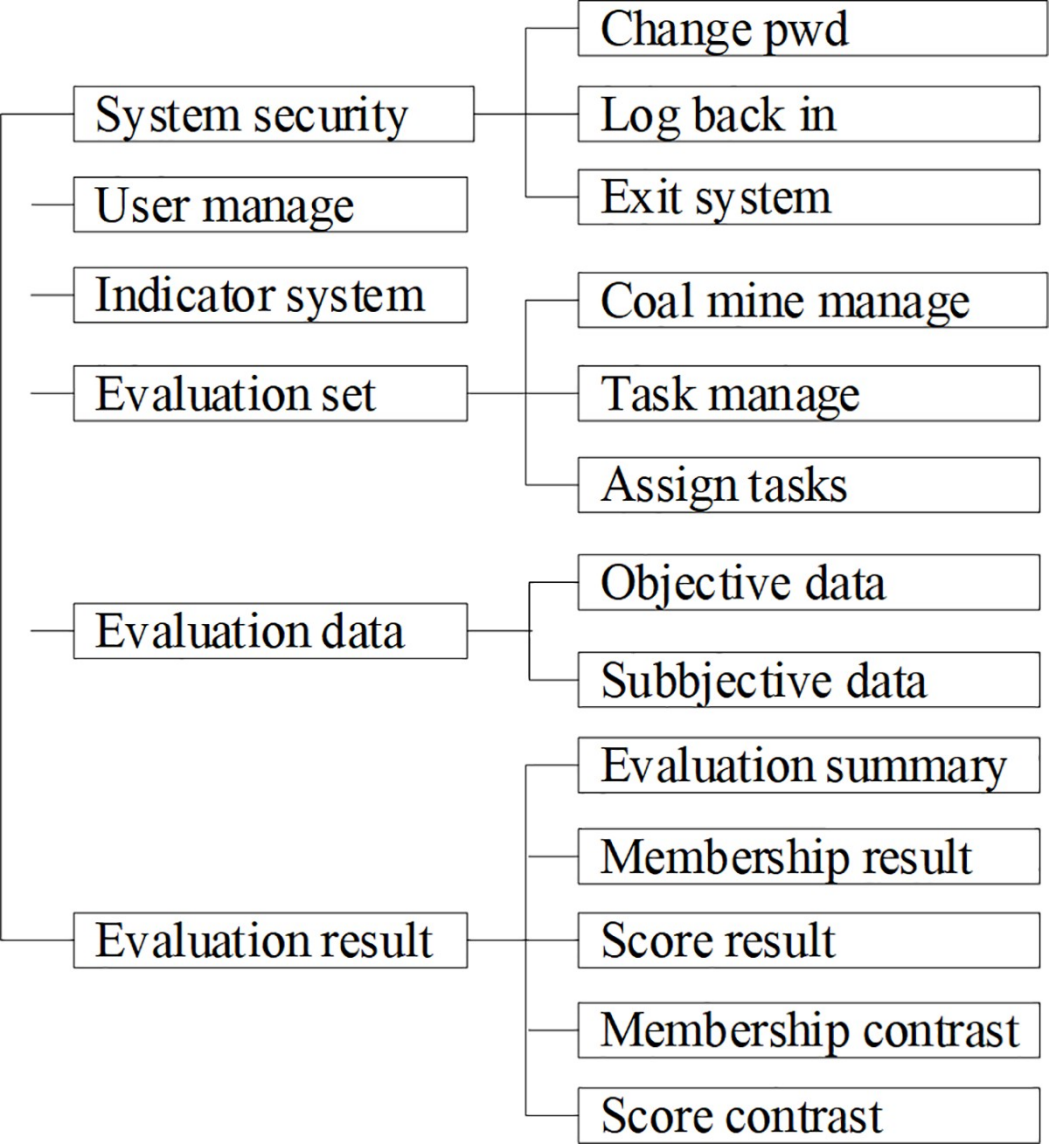
Function modules of Group administrator.

**Fig 4:**
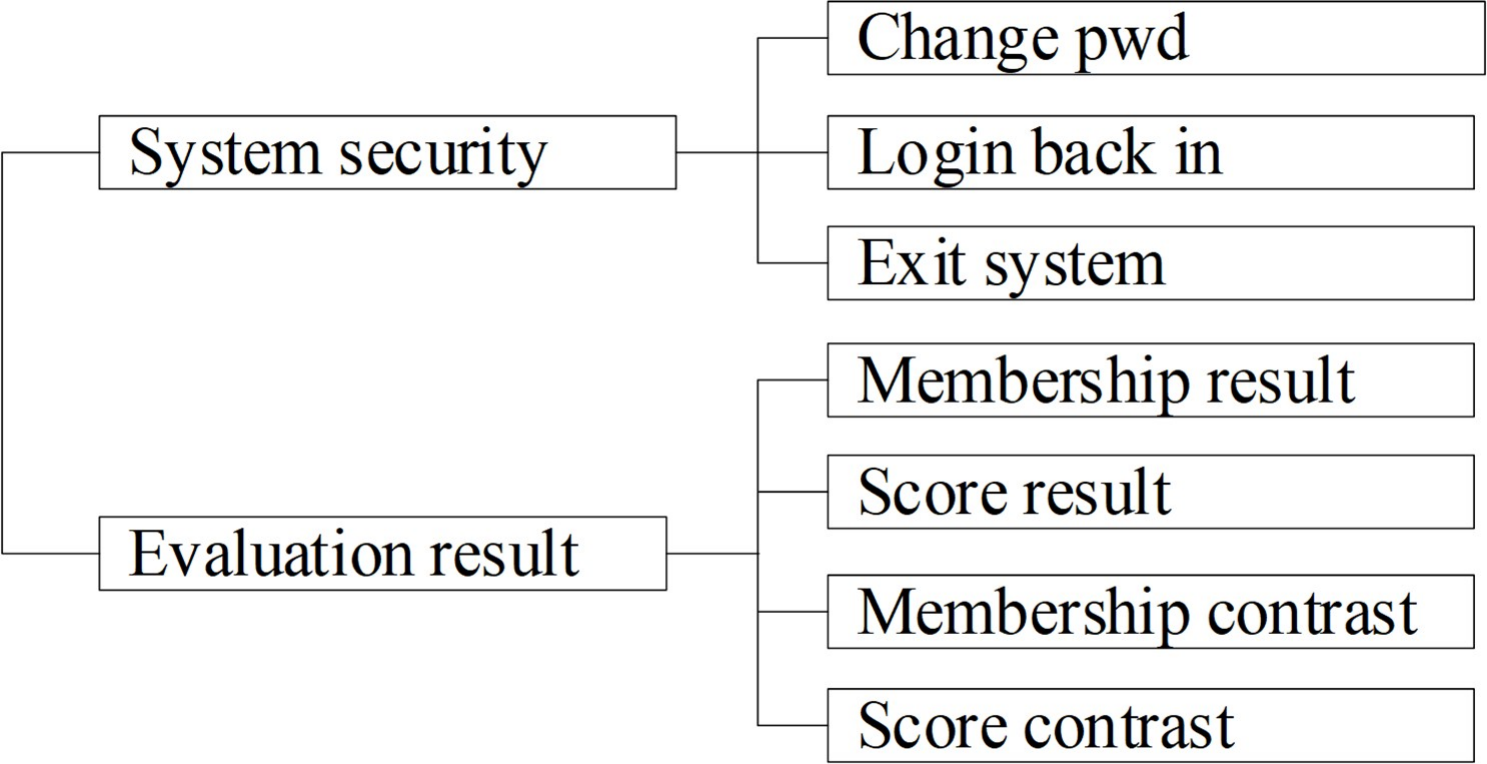
Function modules of Mine administrator.

**Fig 5:**
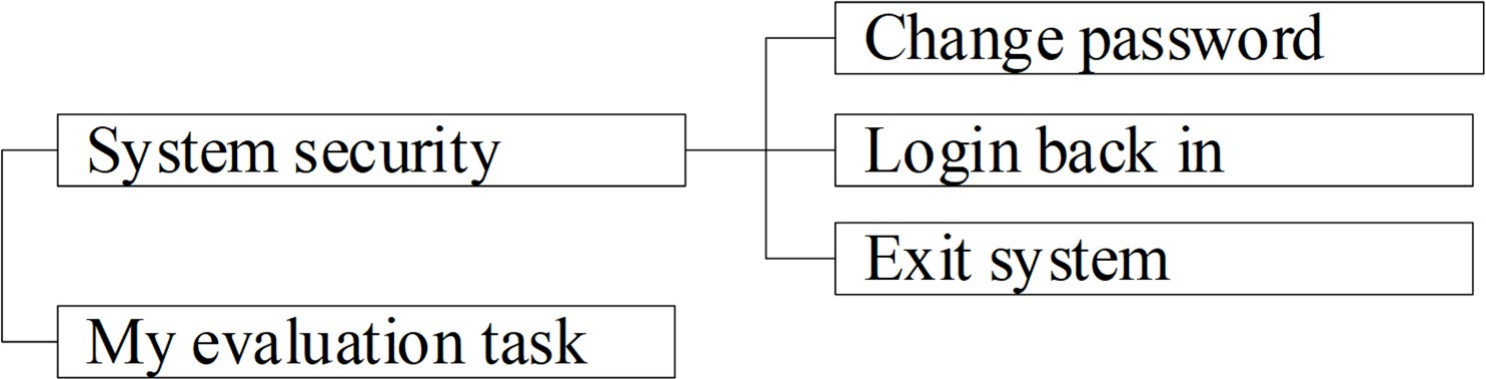
Function modules of expert.

There are three kinds of identities in the system which are Group administrator, Mine administrator and Expert. The function modules of Group administrator are shown in Fig 3. T The function modules of Mine administrator are shown in Fig 4. The function modules of Group administrator are shown in Fig 5.

### 4.2 Online evaluation process design

1. Preparations: The Group administrator logs in the system. Add evaluation experts through the "User manage" module. Establish one or more evaluation indicator systems through the “Indicator system” module which includes indicator system name management, indicator management, objective indicator setting and indicator system inspection. Add evaluated coal mines through the "Coal mine manage" module. Add evaluation tasks through the "Task manage" module which includes specifying the evaluated coal mine, specifying the evaluation indicator system, setting evaluation start time and end time. Assign evaluation tasks to experts through the "Assign tasks" module. Among them, the "indicator management" module provides the form of tree-shaped to add, modify the indicators and set their weights; the "objective indicator setting" module is used to set specific parameters of objective indicators, including characteristics, target, *αA*, *αB*, *αC*, *αD*, *αE*, *Ah*, *Bh*, *Ch*, *Dh*, *Eh*, etc.; the "indicator system inspection" module is used to check whether the sum of the weights of the sub-indicators of each non-final level indicator is 1, and whether the parameters of each objective indicator meet the requirements. If the result of inspection is “Y”, the indicator system is effective; otherwise, it is invalid. Only the "effective" indicator systems can be used in the "Task manage" module, so as to ensure the effectiveness of the evaluation.
2. Evaluation: After the preparations, the Expert logs in the system. View the list of evaluation tasks assigned by the Group administrator through the "My evaluation task" module. Enter the evaluation page to evaluate the finial-level indicators through the "objective indicator evaluation" and "subjective indicator evaluation" sub-module. For each final-level subjective evaluation indicator, check its explanation, choose grade A, B, C, D or E according to his own subjective judgment. For the final-level objective evaluation indicator, check its explanation, accurately input the numeric value of the indicator as required. In particular, for the hoping-have or hoping-no indicator, 1 or 0 should be input, where 1 represents "have" and 0 represents "no".
3. Evaluation data management: After collecting the evaluation data, the Group administrator logs again in the system. Set the status of the evaluation task as "over" through the "Task manage" module to prohibit further evaluation. View the evaluation data through the "Evaluation data" module. If the number of evaluation data is insufficient, incomplete, or any of the evaluation data are obviously unreasonable, he can reset the status of the evaluation task as "not over" through the "Task manage" module, urge relevant experts to supplement or modify the evaluation data, or assign evaluation tasks to other experts to evaluate through the "Assign tasks" module. This process is repeated until the evaluation data collected is sufficient, complete and reasonable. In this condition, set the status of the evaluation task as "over".
4. Evaluation summary: After the completion of the evaluation, get the membership vector, grade and score of each indicator and the evaluated coal mine through the "Evaluation Summary" module.
5. Query of evaluation results: After the evaluation summary, the Group administrator or Mine administrator can login in the system to view the evaluation result. The Group administrator can view the evaluation results of each coal mine under his jurisdiction through the modules of "Membership result" and "Score result". The Mine administrator can view the evaluation results of his own coal mine through the modules of "Membership result" and "Score result".
6. Evaluation result comparison: After the evaluation summary, the Group administrator or Mine administrator can make some comparison through the modules of "Membership comparison” and “Score comparison”. The Group administrator can make comparison of each evaluated coal mine between different evaluations which are evaluated by the same indicator system at different periods (Longitudinal comparison), and make comparison of selected evaluated coal mines which are evaluated by the same indicator system at the same period (Horizontal comparison). The Mine administrator can make comparison of his own coal mine between different evaluations which are evaluated by the same indicator system at different periods (Longitudinal comparison).

### 4.3 Database design

Taking SQL Server as the database management system and following the standardized design principle, the database of the evaluation system is designed.

#### (1) Database structure design

**Fig 6:**
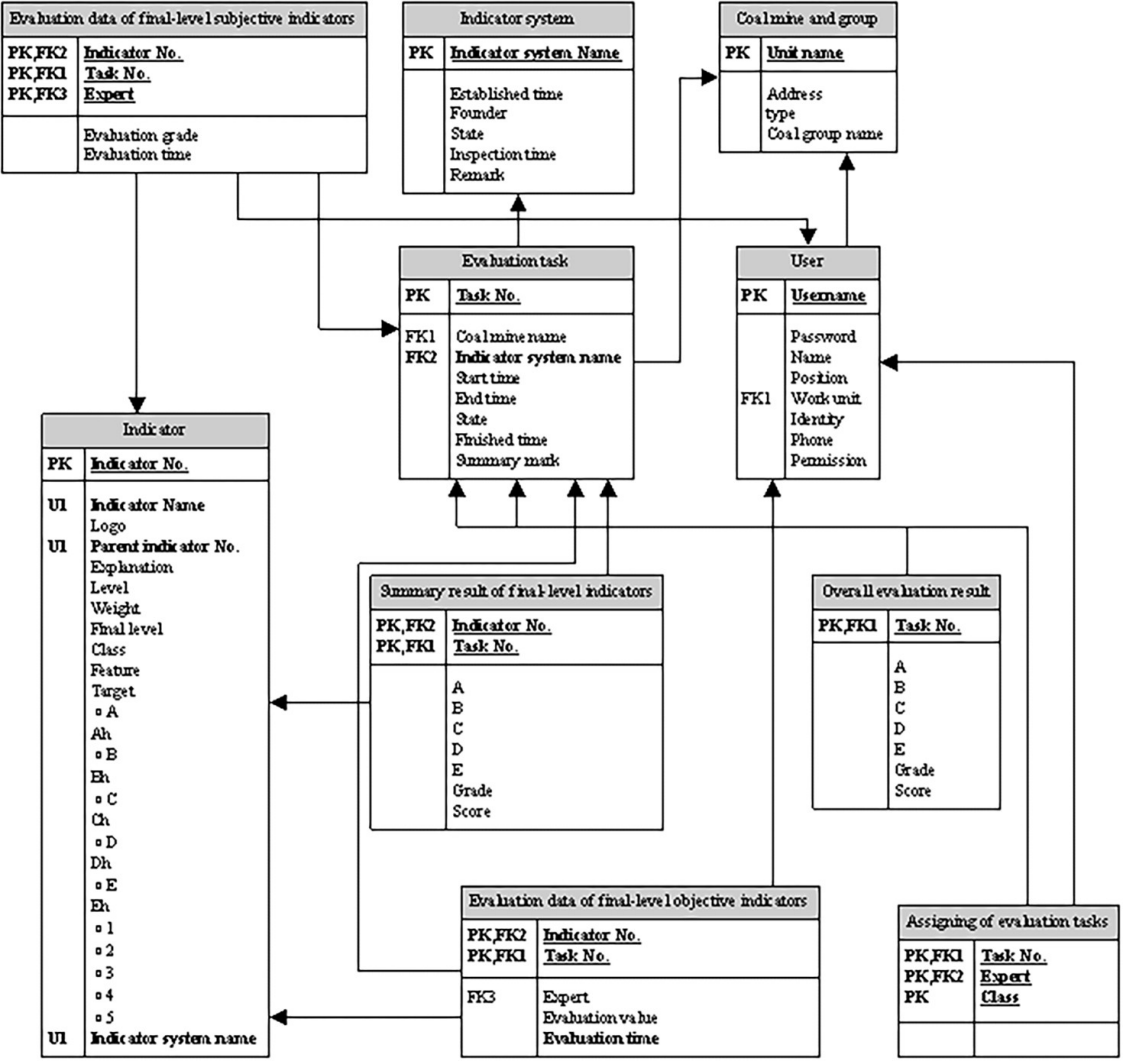
E-R diagram.

The E-R diagram of the database is shown in Fig 6. Set "cascade" to the update rule between the primary table and the child table so as to guarantee data integrity. Set "do nothing" to the delete rule between the primary table and the child table to guarantee data security.

The table “Coal mine and group” used to store the information of coal mines and Coal groups. The field type has two kinds of value which are “Coal mine” and “Coal group”.

The table “User” is used to store the users of the system. The default value of the field “Permission” is “available”. If a user leaves or cannot continue to use the system for some reasons, set "disable" to it. There are three identities for the users in the system, which are Group administrator, Mine administrator and Expert. Among them, the user of Group administrator and Expert comes from one of the Group companies, while the user of Mine administrator comes from one of the coal mines.

The table “Indicator system” is used to store information of the indicator systems. The default value of field "Status" is "unchecked". After system inspection, if an indicator system is valid, set "valid" to it, otherwise set "invalid" to it.

The table “Indicator” is used to store information of the indicators. It has a special structure. When adding a first-level indicator to an indicator system through the system, set 0 to the field “Parent indicator No.”. When adding a second-level indicator to an indicator with the Indicator No. *x*, set *x* to the field “Parent indicator No.”. When adding a third-level indicator to a second-level indicator with the Indicator No. *y*, set *y* to the field “Parent indicator No.”. And so on. It can be seen that each indicator should be set a field “Indicator system name”. In the system, a first-level indicator is led out by the system name, the second-level indicators of each first-level indicator is led out by the field “Parent indicator No.”. And so on. For each first-level indicator, an inverted tree can be established through “recursive process”. All inverted trees for all of the first-level indicators can form a tree-shaped indicator system. By the special structure, the table "Indicator" can store contents of all indicators of infinite levels. The default value of the field “Final level” is “Y”. When adding a sub-indicator to a parent indicator, set “N” to the field of the parent indicator. The value of the field “Class” is Subjective, Objective or “”, which should be specified by the user through the system. If an indicator is a final-level indicator, set "subjective" or "objective" to it, otherwise set "subjective" or "objective" or “” to it according to the specific condition. The field "Feature" is only specific for the final-level objective indicators. For the final-level objective indicators, set one of “hoping-large”, “hoping-target”, “hoping-small”, “hoping-have”, “hoping-no” to it, otherwise set NULL to it. The field “Target” is only specific for the final-level objective hoping-target indicators, that means for other indicators, set NULL to it. The fields “*αA*”, “*αB*”, “*αC*”, “*αD*”, “*αE*”, “*Ah*”, “*Bh*”, “*Ch*”, “*Dh*”, “*Eh*” are only specific for the final-level objective indicators of hoping-large, hoping-target or hoping-small type. These fields are used to calculation the adjustment coefficients *α*1, *α*2, *α*3, *α*4, *α*5. The fields “*α*1”, “*α*2”, “*α*3”, “*α*4”, “*α*5” are the parameters needed to determine the membership degree of an objective indicator of hoping-large, hoping-target or hoping-small type by the Cauchy membership function.

The table “Evaluation task” is used to store information of the evaluation tasks. Among them, the fields “Coal mine name”, “Indicator system name” and “Start time” are defined as the unique index to prevent the same record from being input repeatedly. The default value of the field “status” is “not over”. When the evaluation is over, set “over” to it. The default value of the field "Summary mark" is "N". When the evaluation data is summarized, set “Y” to it.

The table “Assigning of evaluation tasks” is used to assign evaluation tasks to evaluation experts. Where, the field "Class" takes the value of "Objective" or "Subjective".

The table “Evaluation data of final-level objective indicators” is used to store the evaluation data of final-level objective indicators. The fields "Indicator No." and "Task No." are defined as the compound primary key so as to ensure that the same final-level objective indicator of the same evaluation task is only evaluated once. The type of field "Evaluation value" is defined as "real" to ensure that evaluation value of the objective indicators of "hoping-large", "hoping-target”, “hoping-small”, “hoping-have” or “hoping-no” type can be stored by it.

The table “Evaluation data of final-level subjective indicators” is used to store the evaluation data of final-level subjective indicators. Different from table “Evaluation data of final-level objective indicators”, here the fields "Indicator No.", "Task No." and “Expert” are defined as the compound primary key. By this means, the same subjective indicator of the same evaluation task can be evaluated more than once. The value of field "Evaluation grade " is one of "A", "B", "C", "D", and "E".

The table “Summary result of final-level indicators” is used to store the membership degree, grade and score of the indicators obtained by evaluation summary. Among them, fields "A", "B", "C", "D" and "E" are used to store the membership degree of the indicators; field "Grade" is used to store the grade of the indicators determined according to the membership degree of the indicators; field "Score" stores the score calculated by the 5-point system according to the membership degree of the indicators.

The table “Overall evaluation result” is used to store the general evaluation result of each coal mine. The role of each field is the same as that of the table “Summary result of final-level indicators”. The relation between this table and table “Evaluation task” is one-to-one. In theory, they can be merged into one table. However, from the process of the system, it makes more sense to design them separately.

#### (2) Trigger design

For the table “Indicator”, design the triggers shown in Table 2. Among them, the trigger “Indicator_insert” is used to calculate the fields “*α*1”, “*α*2”, “*α*3”, “*α*4”, “*α*5”; trigger “Indicator_update” is used to update the fields “*α*1”, “*α*2”, “*α*3”, “*α*4”, “*α*5”. Triggers “Indicator_noinsert”, “Indicator_noupdate”, “Indicator_nodelete” are used to ensure the validity of the evaluation data and results.

**Table 2:** Trigger.

No.	Name	Role	Content
1	Indicator_insert	Calculate the fields “*α*1”, “*α*2”, “*α*3”, “*α*4”, *α*5” by the fields “*αA*”, *αB*”, “*αC*”, “*αD*”, “*αE*”, “*Ah*”, *Bh*”, “*Ch*”, “*Dh*”, “*Eh*” when an indicator is inserted and the field “Feature” is hoping-large, hoping-target or hoping-small	S1 Appendix
2	Indicator_update	Recalculate the fields “*α*1”, “*α*2”, “*α*3”, “*α*4”, *α*5” by the fields “*αA*”, *αB*”, “*αC*”, “*αD*”, “*αE*”, “*Ah*”, *Bh*”, “*Ch*”, “*Dh*”, “*Eh*” When one of the fields “*αA*”, *αB*”, “*αC*”, “*αD*”, “*αE*”, “*Ah*”, *Bh*”, “*Ch*”, “*Dh*”, “*Eh*” is modified and the field “Feature” is hoping-large, hoping-target or hoping-small	S2 Appendix
3	Indicator_noinsert	Prevent an indicator from being inserted, when the indicator system to which this indicator belongs has been used to evaluate any coal mines (Any indicators of this indicator system appears in the table "Evaluation data of final-level subjective indicators" or "Evaluation data of final-level subjective indicators")	Not listed
4	Indicator_noupate	Prevent an indicator from being updated, when the indicator system to which this indicator belongs has been used to evaluate any coal mine	Not listed
5	Indicator_nodelete	Prevent an indicator from being deleted, when the indicator system to which this indicator belongs has been used to evaluate any coal mine	Not listed

#### (3) Stored procedure design

It can be seen from the system evaluation process described in section 4.2 that the work with the largest amount of calculation in the system is the summary of evaluation results. In order to simplify the front-end program, design a stored procedure named “Summary by Task No.” with the parameter “@taskno" to realize the evaluation summary. It firstly uses the “cursor”, “while loop” to determine the membership vector, grade and score of the final-level indicators from tables “Evaluation data of final-level objective indicators” and "Evaluation data of final-level subjective indicators” by the parameter evaluation task “@taskno” based on the step (3) described in section 3, then determines the membership, grade and score of the non-final-level indicators and the evaluated coal mine from low level to high level respectively, finally stores them into the data tables “Summary result of indicators” and “Overall evaluation result”. The specific code is shown in S3 Appendix.

### 4.4 Program design

Taking C#.NET as the development language, a set of online evaluation system of coal mine comprehensive level base on FCE is designed and developed. The specific design is not described in this paper. Among it, the program calls the stored procedure “Summary by Task No.” to get summary result of evaluation.

## 5. Case study

Taking Coal group PM as an example, five coal mines under its jurisdiction are evaluated for test.

**Fig 7:**
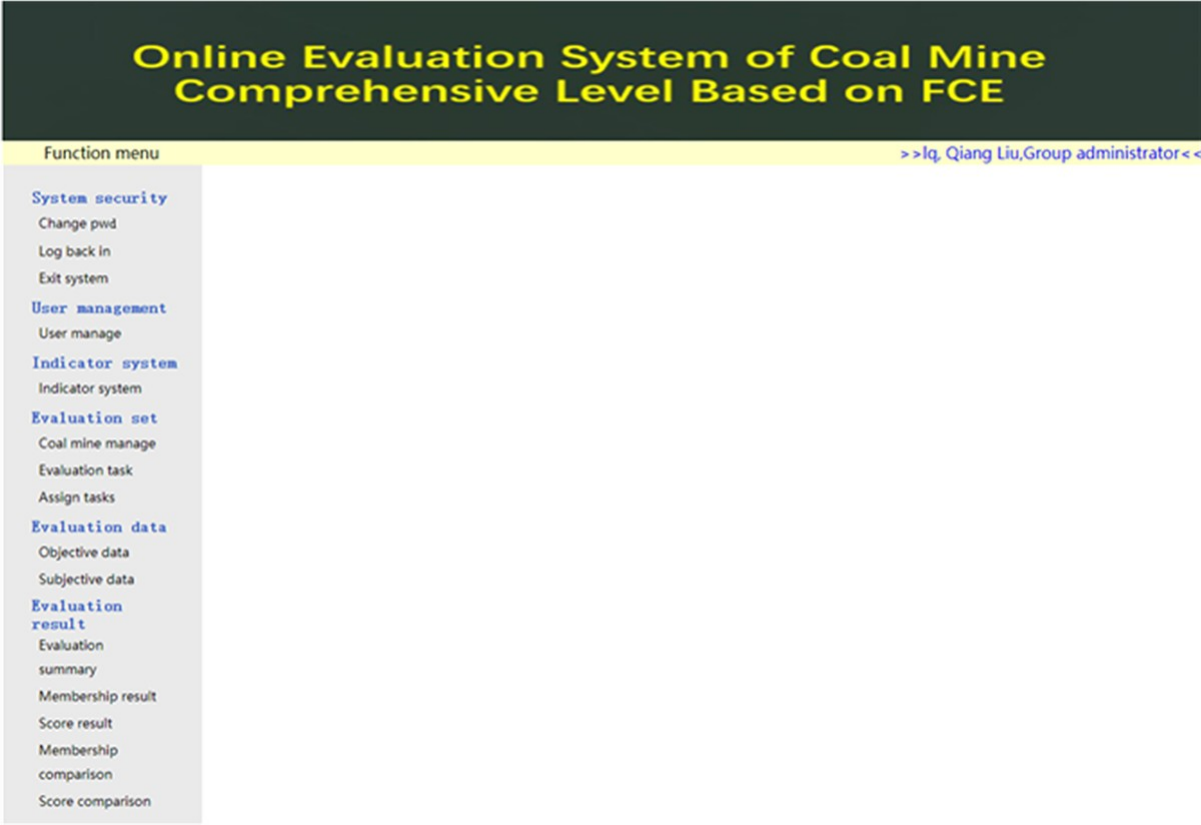
Main interface of Group administrator.

Fig 7 is the main interface of Group administrator (username: lq). The main interfaces of Mine administrator (username: zw) and Expert (username: lxs) are not shown in this paper.

**Fig 8:**
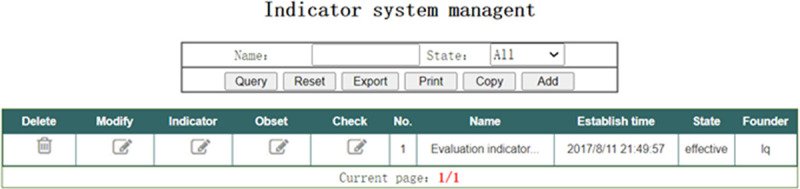
Indicator system management.

In the interface of “Indicator system management” shown in Fig 8, the Group administrator can add or delete the indicator system, set contents of indicators for the indicator system, set parameters for the final-level objective indicators, and check the indicator system. In order to ensure the effectiveness of the evaluation, before modifying or deleting the indicator system, the trigger designed in the database will check whether the indicator system has been used to evaluate any coal mines. Once used, modification or deletion is not allowed.

**Fig 9:**
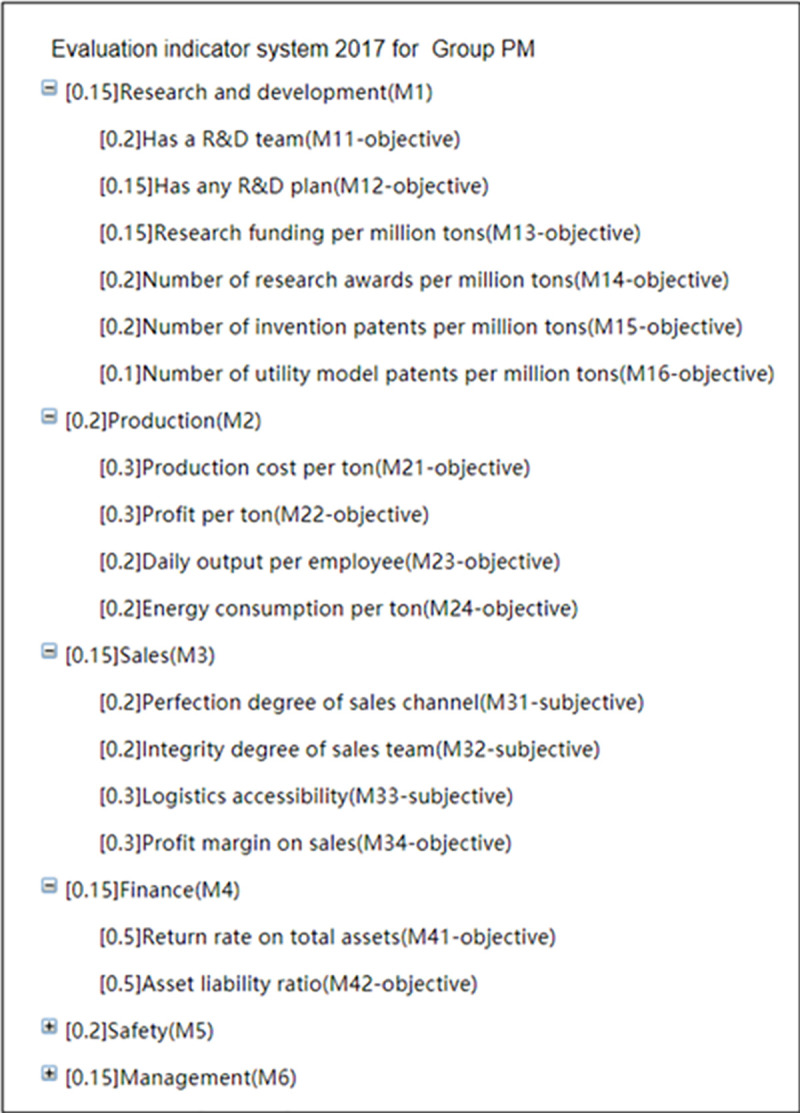
Indicator management.

In the interface of “Indicator management” shown in Fig 9, the Group administrator can click on the indicator system name to add first-level indicators for it, or click on any indicator to add sub-indicators for it, modify it or delete it.

**Fig 10:**
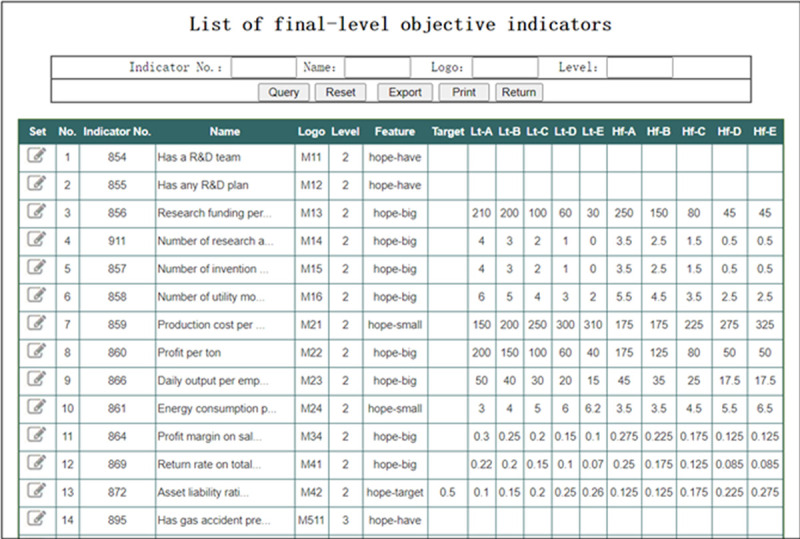
List of final-level objective indicators.

**Fig 11:**
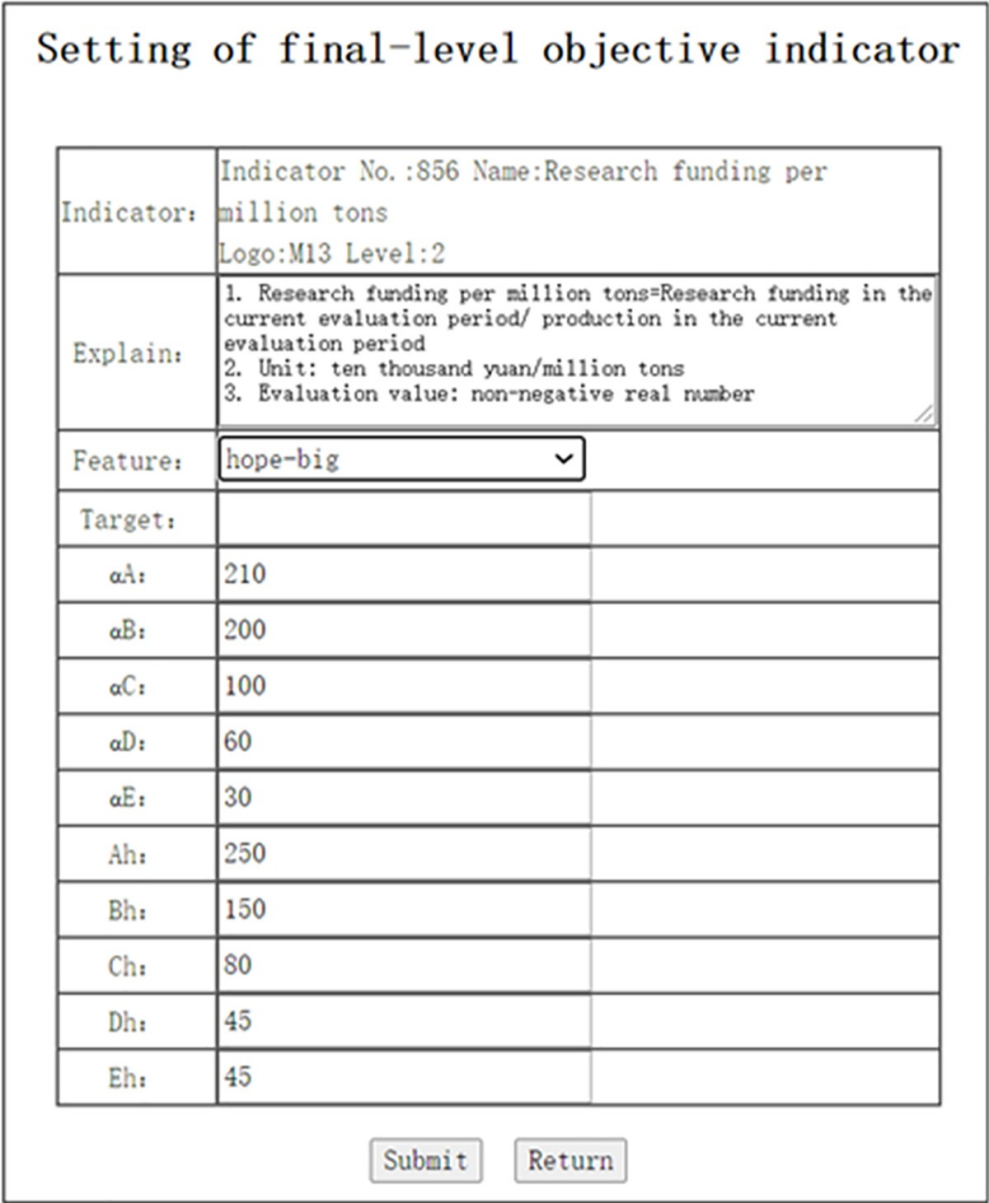
Setting of final-level objective indiator (Indicator No.: 856).

In the interface of “Indicator system management” shown in Fig 8, click the button “Obset” to enter the interface as shown in Fig 10. This interface lists all of the final-level objective indicators of the indicator system. Click the button “Set” in column 1 to enter the interface shown in Fig 11. In this interface, the parameters of the objective evaluation indicator can be set. Fig 11 shows the parameter setting interface with the Indicator No. 856.

**Fig 12:**
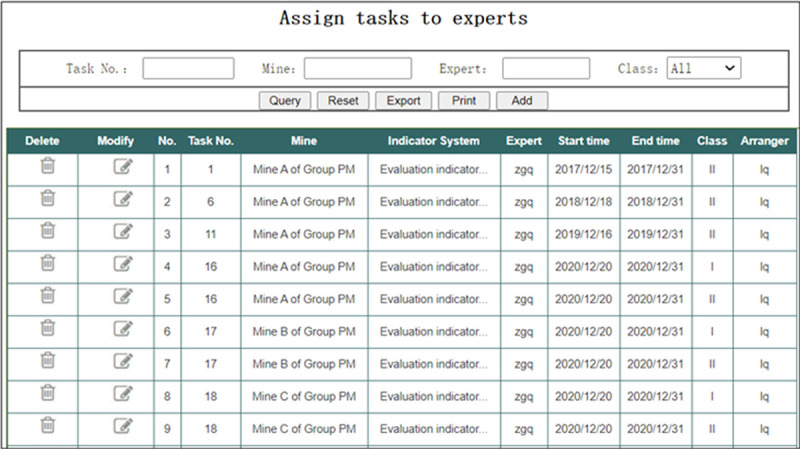
Assigning tasks to experts.

In the interface of “Assigning tasks to experts” shown in Fig 12, the Group administrator can assign tasks to evaluation experts. It should be pointed out that because each evaluation expert has his own expertise, it is not necessary for each evaluation expert to evaluate all of the objective indicators and subjective indicators, but to make reasonable arrangements according to their expertise. For example, some evaluation experts are responsible for evaluation of the objective indicators, and some experts are responsible for evaluation of the subjective evaluation indicators, and some experts are responsible for evaluation of both objective and subjective indicators.

**Fig 13:**
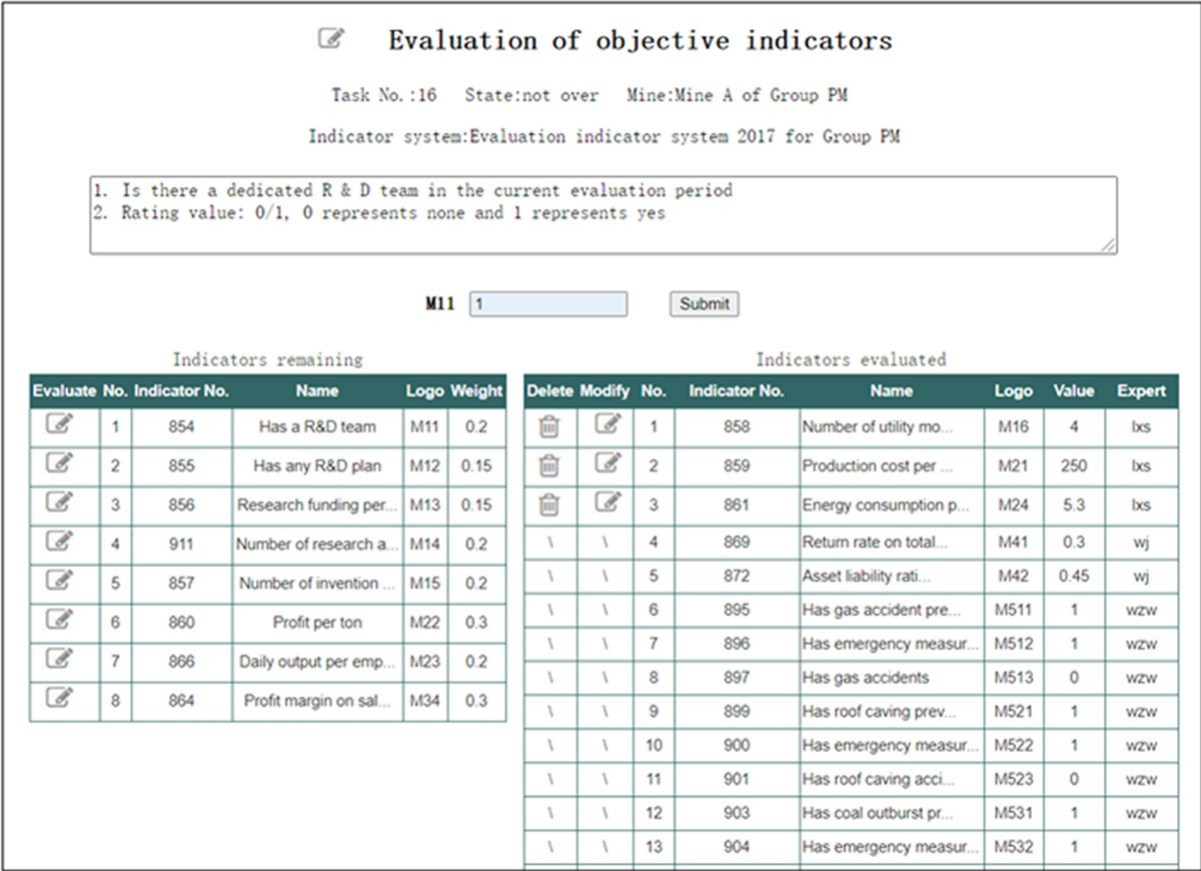
Evaluation of final-level objectvie indicators.

In the interface of “Evaluation of final-level objectvie indicators” shown in Fig 13, the Expert can select the objective indicators he is familiar with and input or modify the evaluation value of these indicators according to their explanation. It can be seen that current Expert cannot modify or delete evaluation data given by other Experts.

**Fig 14:**
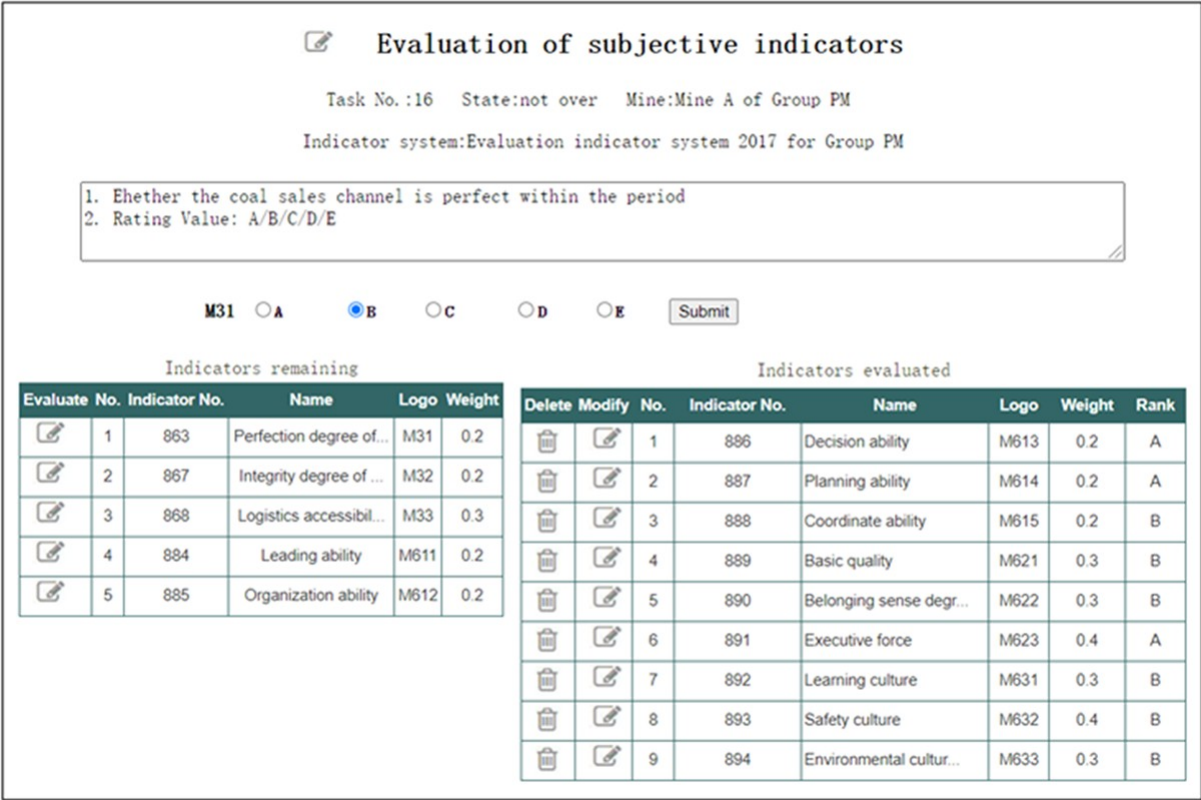
Evaluation of final-level subjective indicators.

In the evaluation interface of “Evaluation of final-level subjective indicators” shown in Fig 14, the Expert can choose the familiar subjective evaluation indicators to give their grades.

**Fig 15:**
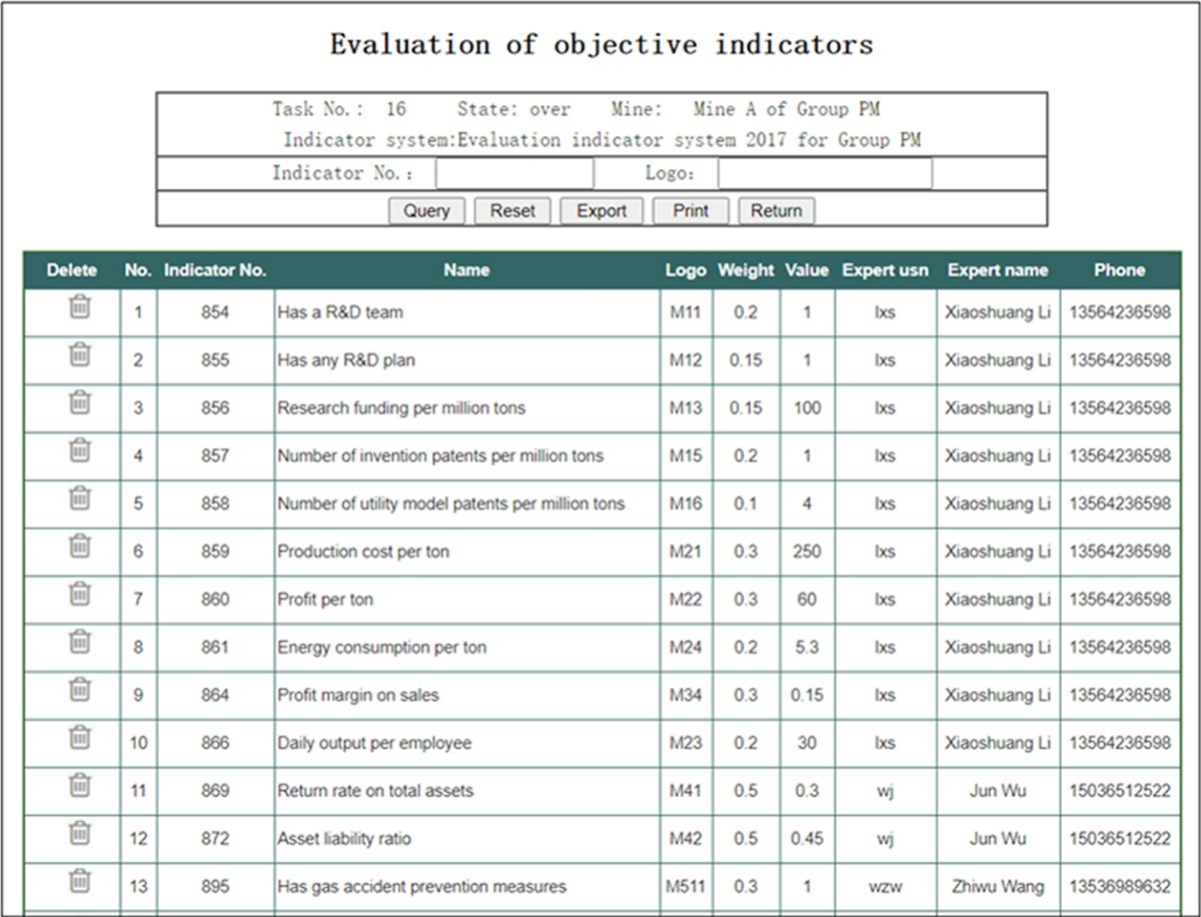
Evaluation data of final-level objective indicators.

In the interface of “Evaluation data of final-level objective indicators” shown in Fig 15, the Group administrator can view the evaluation data of final-level objective indicators given by all of the Experts. On the one hand, he can check whether the evaluation data are complete. On the other hand, he can check whether the evaluation data are reasonable, and give some human intervention when necessary.

**Fig 16:**
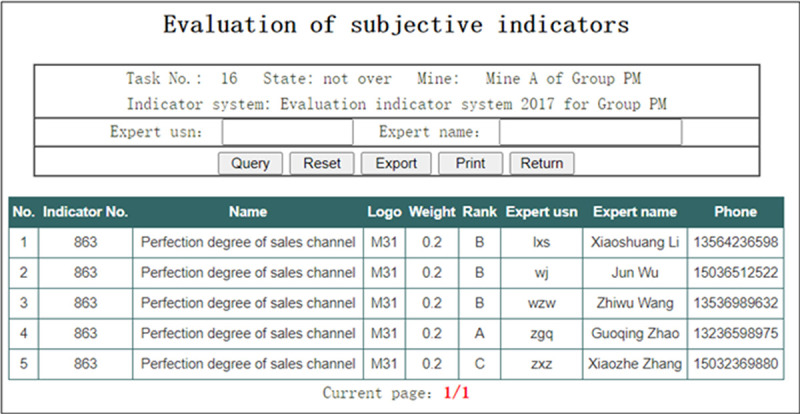
Evaluation data of final-level subjective indicators.

In the interface of “Evaluation data of final-level subjective indicators” shown in Fig 16, the Group administrator can view the evaluation data of final-level subjective indicators given by all of the Experts.

**Fig 17:**
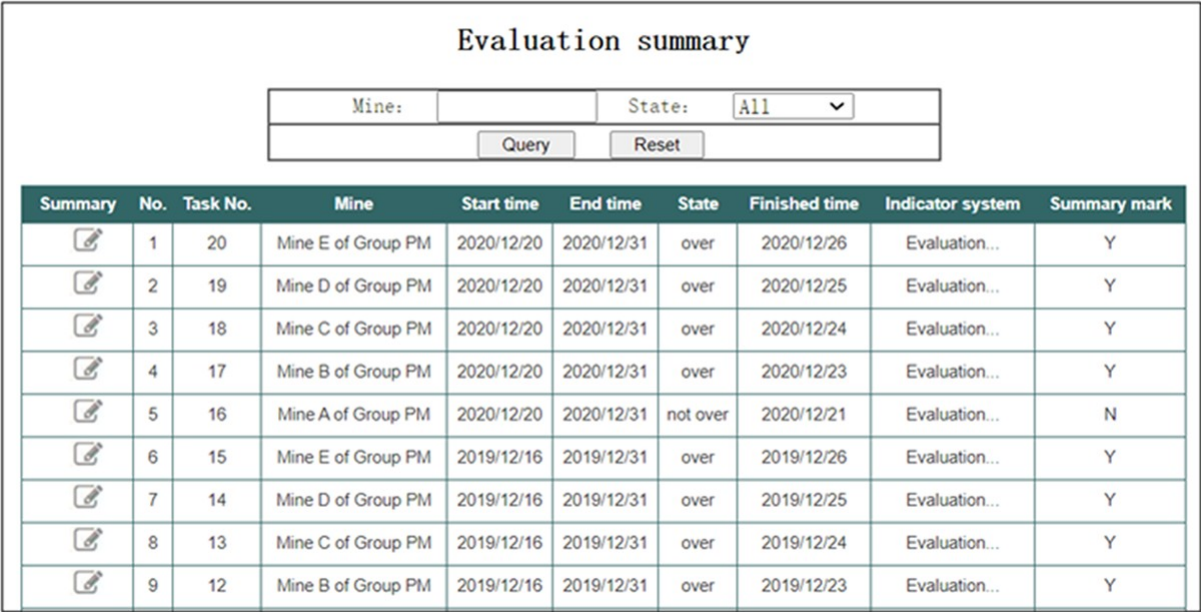
Evaluation summary.

In the interface of “Evaluation summary” shown in Fig 17, the Group administrator can summarize each evaluation task in turn. For example, click the Summary button of Task No. 16, the system calls the stored procedure “Summary by Task No.” with 16 as the parameter. Wait a moment, the Summary is completed, and the summary results are stored into the data tables “Summary result of indicators” and “Overall evaluation result”.

**Fig 18:**
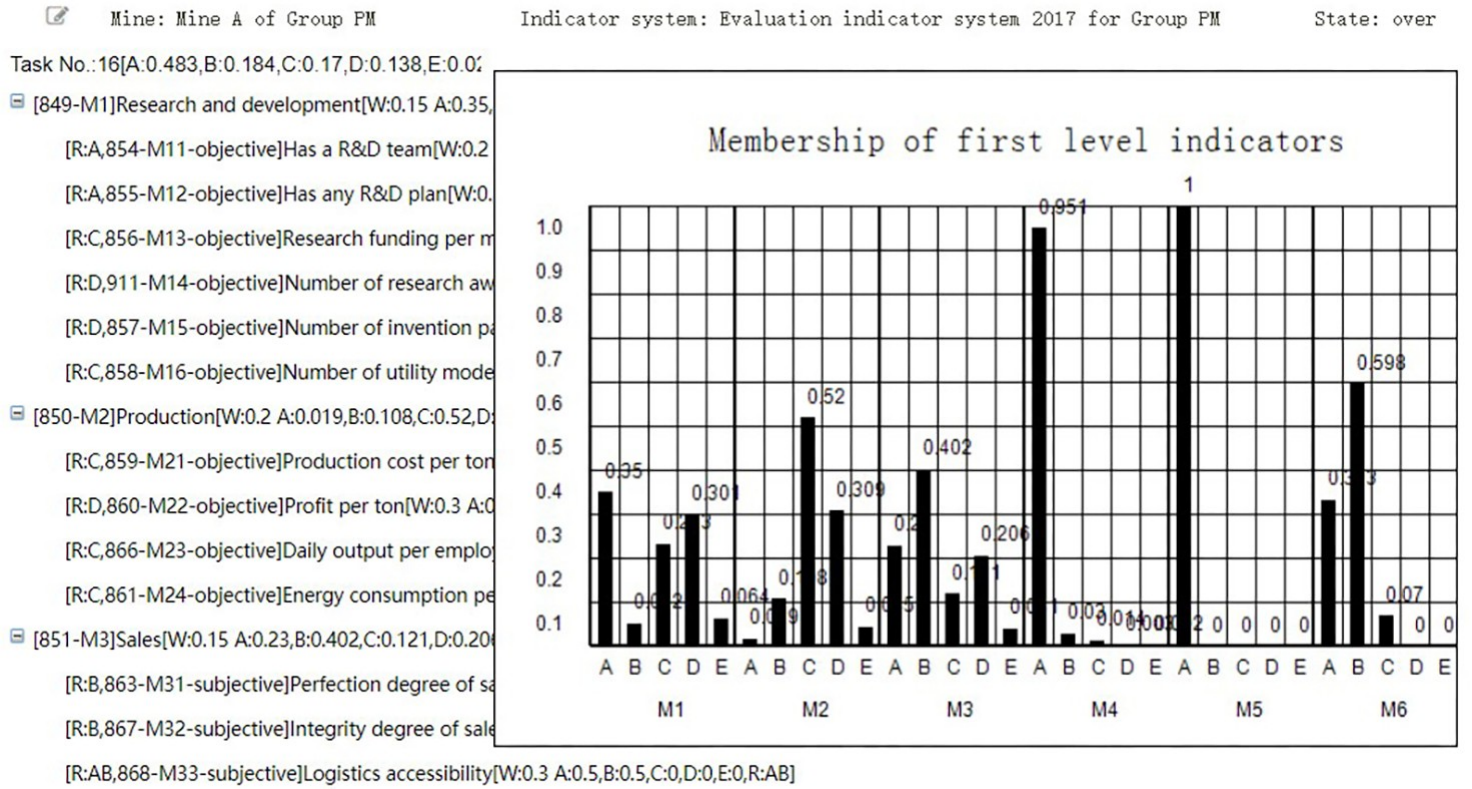
Membership evaluation result.

In the interface of “Membership evaluation result” shown in Fig 18, the Group administrator can view the membership degree of each indicator or the evaluated coal mine. He can also click the first row to view the membership degrees of each first-level indicators, or click the non-final-level indicator to view the membership degrees of its sub-indicators.

**Fig 19:**
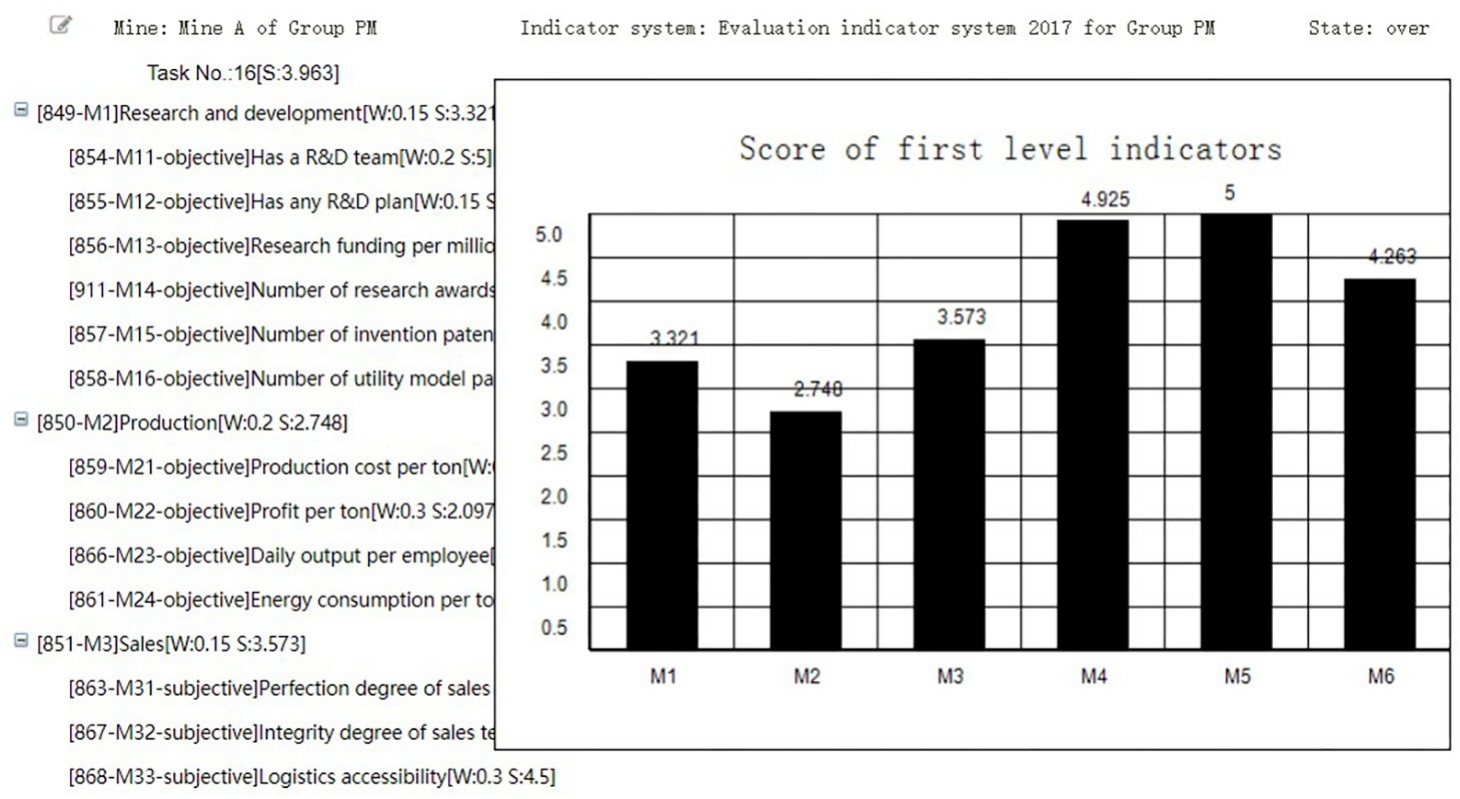
Score evaluation result.

In the interface of “Score evaluation result” shown in Fig 19, the Group administrator can view the score of each indicator or the evaluated coal mine. He can also click the first row to view the scores of each first-level indicators, or click the non-final-level indicator to view the scores of its sub-indicators.

**Fig 20:**
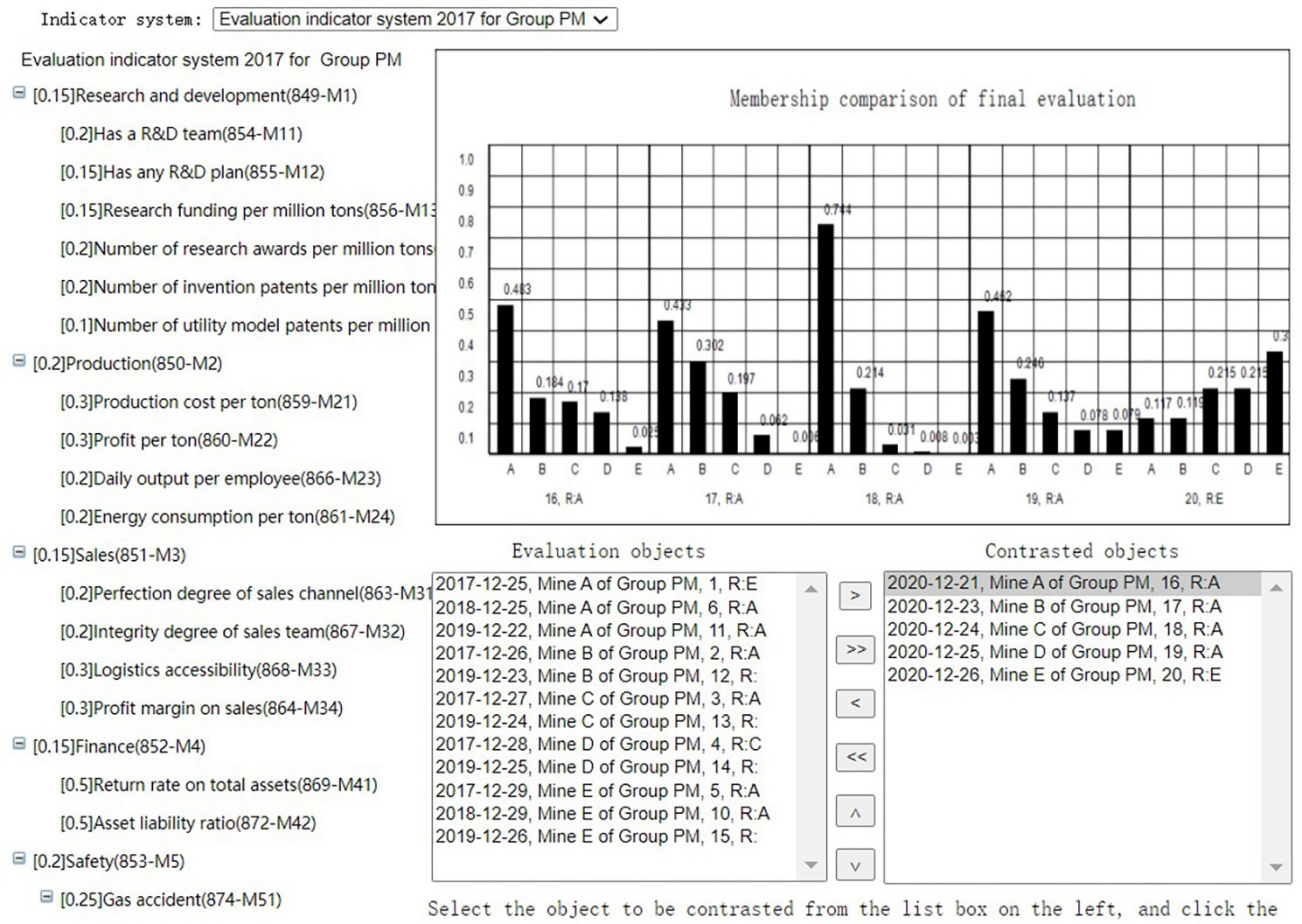
Horizontal membership comparison.

**Fig 21:**
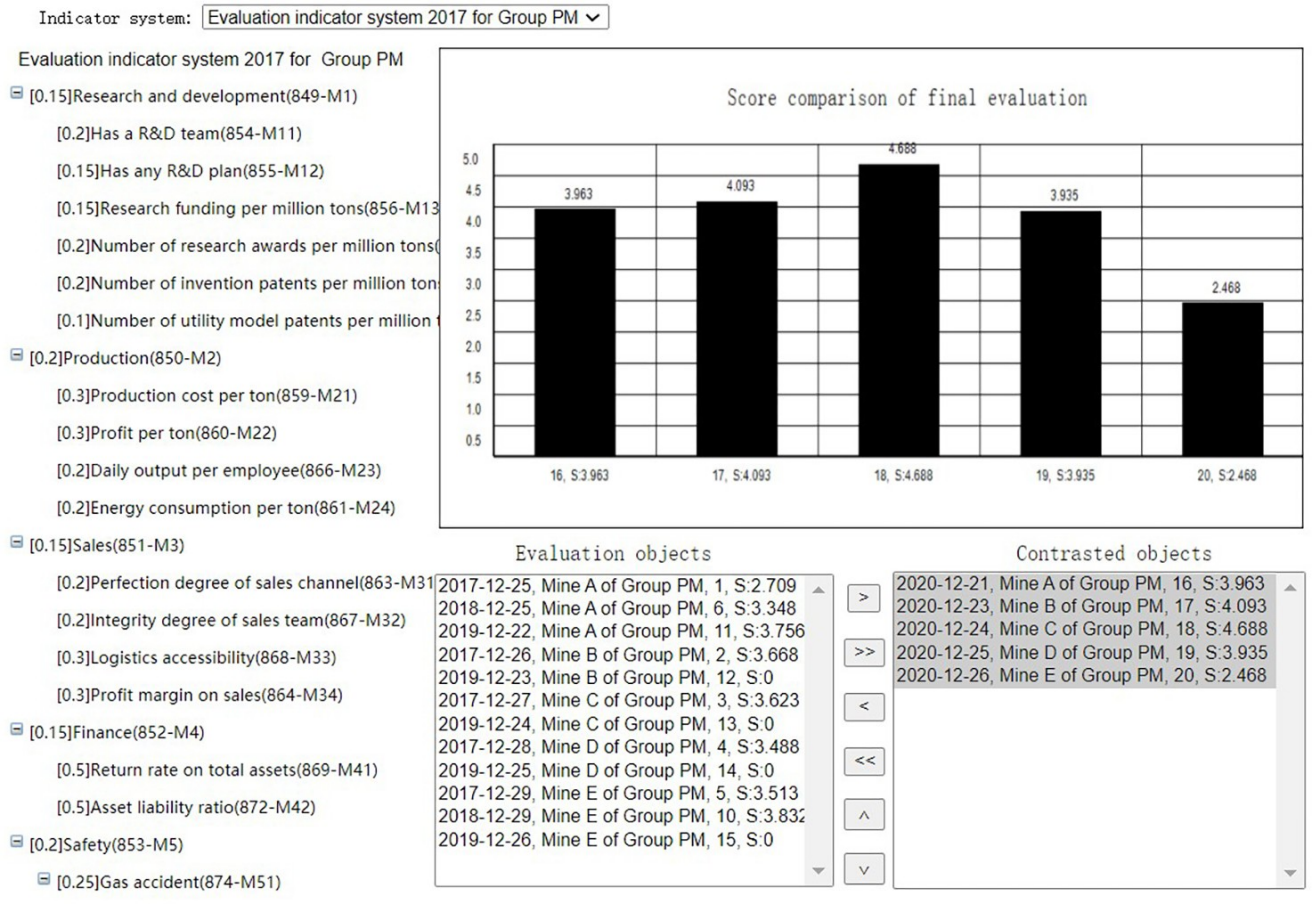
Horizontal score comparison.

**Fig 22:**
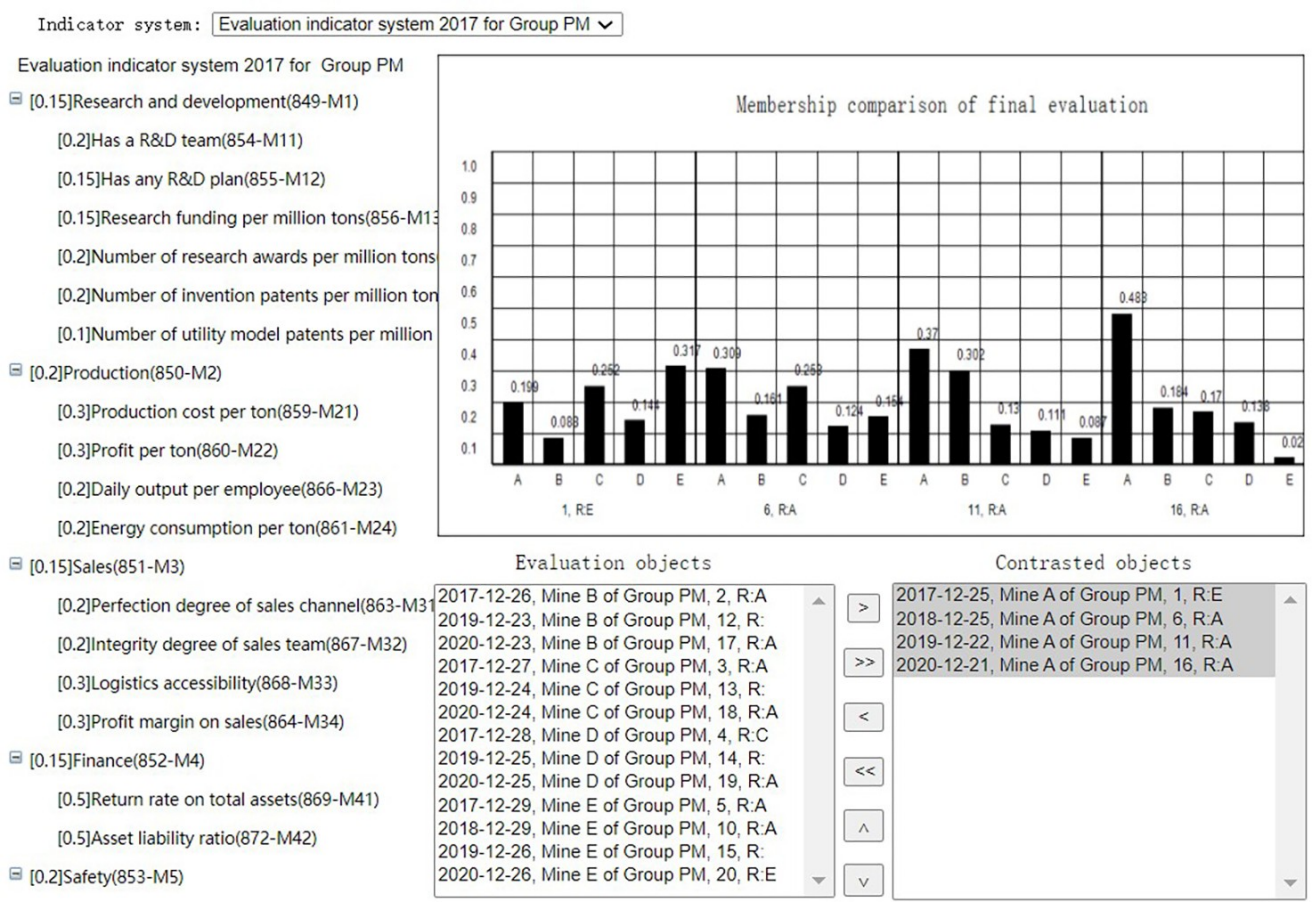
Longitudinal membership comparison.

**Fig 23:**
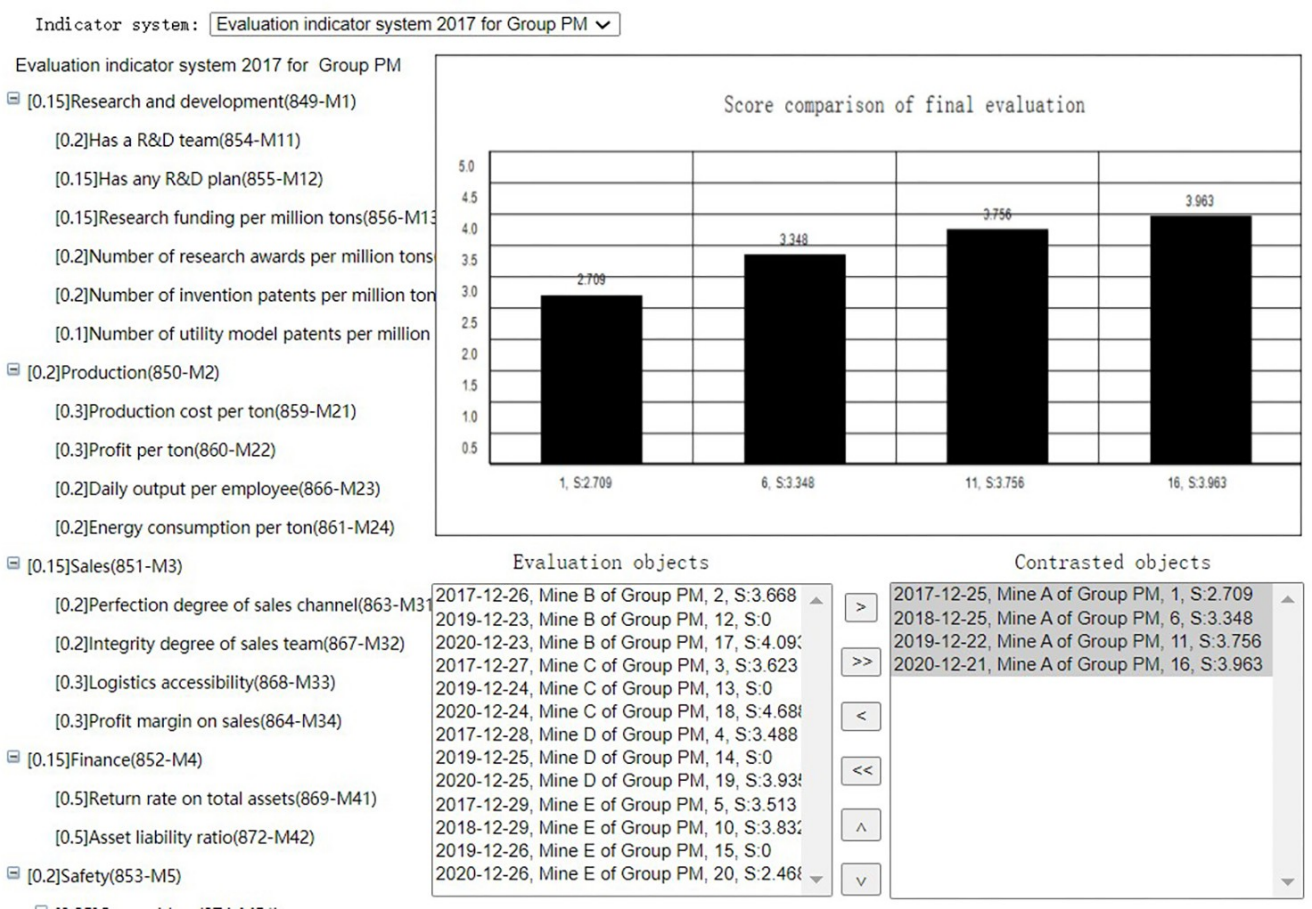
Longitudinal score comparison.

After evaluation summary of each evaluation task, the Group administrator can select the evalations of the coal mines evaluated by the same indicator system at the same period to get the membership comparison or score comparison(Horizontal comparison), as shown in Figs 20 and 21. He can also select the evaluations of the same coal mine evaluated by the same indicator system at different periods to get the membership comparison or score comparison(Horizontal comparison), as shown in Figs 22 and 23. The Mine administrator can select the evalations of his own coal mine evaluated by the same indicator system at different periods to get the membership comparison or get the score comparison(Horizontal comparison), as shown in Figs 22 and 23.

Through comparison, the Mine adminstrator can easily seen the change trend of the evaluation result of the coal mine and each indicator of his own coal mine, and the Group adminstrator can easily seen the advantages and disadvantages of each coal mine under its jurisdiction.

## 6. Conclusion and prospect

Aiming at the coal industry group, an online evaluation method of coal mine comprehensive level based on FCE is put forward. The research conclusions are as follows.

1. According to the comprehensive level of coal mines, a multi-level evaluation indicator system is established from research and development, production, sales, finance, safety and management. Only in a more systematic and comprehensive perspective can the comprehensive level of coal mines be evaluated, so as to ensure the comprehensiveness of the evaluation results.
2. For the final-level objective evaluation indicators (hoping-large, hoping-small, hoping-target, hoping-have, hoping-no) and subjective evaluation indicators, appropriate methods are adopted to determine their membership vectors, and then FCE is adopted to evaluate the coal mines, which expands the application scope of the evaluation method.
3. The online evaluation system of coal mine comprehensive level designed in this paper can make evaluation, summary and comparison of coal mines conveniently and efficiently.
4. For a coal mine, by comparing the evaluation results of the coal mine or each indicator in different periods (horizontal comparison), the change trend can be seen. If there is a good trend, it should be maintained. However, if there is a deterioration trend, improvement measures should be taken to prevent it from continuing to deteriorate. For the Coal group, by comparing the evaluation results of different coal mines and each indicator in the same period (longitudinal comparison), the advantages and disadvantages of each coal mine can be seen. For the disadvantages, the Coal group can timely urge the coal mine to improve them so as to improve the competitiveness of the coal mine and the Coal group.
5. The method proposed in this paper can not only be used to evaluate coal mines, but also to evaluate similar enterprises or organizations after a little modification.

It should be pointed that the method proposed in this paper is suitable for the evaluation of subjective indicators or the combination of subjective and objective indicators. If all of the evaluation indicators are objective indicators, other accurate quantitative methods may be more suitable, such as Entropy method, FA method and TOPSIS method, etc.

Although the method proposed in this paper has realized the online evaluation of the comprehensive level of coal mines based on FCE, there are two research directions in the next step. One is to give improvement measures on the basis of the evaluation to make the evaluation system more intelligent. The second is to develop mobile online evaluation system (phone APP) so as to make the evaluation more convenient.

## Supporting information

S1 Appendix Trigger: Indicator insert. (DOCX)

S2 Appendix Trigger Indicator update. (DOCX)

S3 Appendix Procedure: Summary by Task No. (DOCX)
