# Supplementary material for: Online evaluation method of coal mine comprehensive level based on FCE
Source: PLoS One. 2021 Aug 16;16(8):e0256026. doi: 10.1371/journal.pone.0256026 (PMC8366963; doi:10.1371/journal.pone.0256026)
Supplement: S1 Appendix — (DOCX) [file pone.0256026.s001.docx]

**S1 Appendix**

**Trigger: Indicator_insert**

create trigger Indicator_insert on Indicator after insert as

begin

declare @bh bigint, @feature varchar(50)

declare @αA real, @αB real, @αC real, @αD real, @αE real

declare @aA real, @Ah real, @Bh real, @Ch real, @Dh real, @Eh real

declare @α1 real, @α2 real, @α3 real, @α4 real, @α5 real

select @αA=αA, @αB=αB, @αC=αC, @αD=αD, @αE=αE,@Ah=Ah, @Bh=Bh, @Ch=Ch, @Dh=Dh, @Eh=Eh,@feature=feature,@bh=[Indicator No.] from inserted

if @feature='hoping-large'

set @α1=1/power(@Ah-@αA,-2)

else

set @α1=1/power(@Ah-@αA,2)

set @α2=1/power(@Bh-@αB,2)

set @α3=1/power(@Ch-@αC,2)

set @α4=1/power(@Dh-@αD,2)

if @feature='hoping-large'

set @α5=1/power(@Eh-@αE,2)

else

set @α5=1/power(@Eh-@αE,-2)

update Indicator set α1=@α1,α2=@α2,α3=@α3,α4=@α4,α5=@α5 where [Indicator No.]=@bh

end
