# Supplementary material for: Online evaluation method of coal mine comprehensive level based on FCE
Source: PLoS One. 2021 Aug 16;16(8):e0256026. doi: 10.1371/journal.pone.0256026 (PMC8366963; doi:10.1371/journal.pone.0256026)
Supplement: S3 Appendix — (DOCX) [file pone.0256026.s003.docx]

**S3 Appendix**

**Procedure: Summary by Task No**

create proc [Summary by Task No.] (@taskno bigint) as

begin

declare @bh bigint, @feature varchar(50)

declare @target real, @value real, @x real

declare @αA real, @αB real, @αC real, @αD real, @αE real

declare @aA real, @Ah real, @Bh real, @Ch real, @Dh real, @Eh real

declare @α1 real, @α2 real, @α3 real, @α4 real, @α5 real

declare @sa real, @sb real, @sc real, @sd real, @se real, @s real, @score real

declare @weight real, @level int, @evanum real

declare @A real, @B real, @C real, @D real, @E real, @grade varchar(50), @mld real

delete from [Overall evaluation result] where taskno=@taskno --delete original overall evaluation result

delete from [Summary result of indicators] where taskno=@taskno -- delete original summary result of indicators

**--Step 1: Determine membership of final-level subjective indicators**

declare cursor1 cursor for select [Indicator No.] from [Evaluation task],Indicator where [Evaluation task].[Indicator system name]=Indicator. [Indicator system name] and [Task No.]=@taskno and [Final level]='Y' and Class='subjective'

open cursor1

fetch next from cursor1 into @bh

while @@fetch_status=0

begin

select @evanum=count(*) from [Evaluation data of subjective indicators] where [Task No.]=@taskno and [Indicator No.]=@bh

if @evanum>0 -- There are evaluation data of subjective indicators.

begin

set @score=0

select @sa=count(*)/@evanum from [Evaluation data of subjective indicators] where [Task No.]=@taskno and [Indicator No.]=@bh and [Evaluation grade]='A'

select @sb=count(*)/@evanum from [Evaluation data of subjective indicators] where [Task No.]=@taskno and [Indicator No.]=@bh and [Evaluation grade]='B'

select @sc=count(*)/@evanum from [Evaluation data of subjective indicators] where [Task No.]=@taskno and [Indicator No.]=@bh and [Evaluation grade]='C'

select @sd=count(*)/@evanum from [Evaluation data of subjective indicators] where [Task No.]=@taskno and [Indicator No.]=@bh and [Evaluation grade]='D'

select @se=count(*)/@evanum from [Evaluation data of subjective indicators] where [Task No.]=@taskno and [Indicator No.]=@bh and [Evaluation grade]='E'

end

else -- There are not evaluation data of subjective indicators.

begin

set @sa=0;set @sb=0;set @sc=0;set @sd=0;set @se=0;set @score=0

end

set @score=5*@sa+4*@sb+3*@sc+2*@sd+1*@se –Calculate score

set @mld=dbo.maxlsd(@sa,@sb,@sc,@sd,@se) -- Calculate max membership by custom function “maxsld”

set @grade=''

if @mld>0

begin

if abs(@sa-@mld)<=0.001 set @grade =@grade +'A'

if abs(@sb-@mld)<=0.001 set @grade =@grade +'B'

if abs(@sc-@mld)<=0.001 set @grade =@grade +'C'

if abs(@sd-@mld)<=0.001 set @grade =@grade +'D'

if abs(@se-@mld)<=0.001 set @grade =@grade +'E'

end

insert into [Summary result of indicators]([Task No.], [Indicator No.], A, B, C, D, E, Grade, Score) values(@taskno,@bh,round(@sa,3),round(@sb,3),round(@sc,3),round(@sd,3),round(@se,3),@grade,round(@score,3))

fetch next from cursor1 into @bh

end

close cursor1

deallocate cursor1

--**Step2: Determine membership of final-level objective indicators**

declare cursor1 cursor for select [Indicator No.], Feature, Target, @αA, @αB, @αC, @αE, @αE, α1, α2, α3, α4, α5 from [Evaluation task],Indicator where [Evaluation task].[Indicator system name]=Indicator. [Indicator system name] and [Task No.]=@taskno and [Final level]='Y' and Class='objective'

open cursor1

fetch next from cursor1 into @bh,@feature,@target,@αA,@αB,@αC,@αD,@αE,@α1,@α2,@α3,@α4,@α5

while @@fetch_status=0

begin

select @evanum=count(*) from [Evaluation data of objective indicators] where [Task No.]=@taskno and [Indicator No.]=@bh

if @evanum=1 -- There are evaluation data of objective indicators.

begin

set @score=0

--Calculate membership by Cauchy membership function

select @value=[Evaluation value] from [Evaluation data of objective indicators] where [Task No.]=@taskno and [Indicator No.]=@bh

if @feature='hope-samll'

begin

if @value<=@αA

begin

set @sa=1;set @sb=0;set @sc=0;set @sd=0;set @se=0

end

else if @value<=@αE

begin

set @sa=1/(1+@α1*power(@value-@αA,2))

set @sb=1/(1+@α2*power(@value-@αB,2))

set @sc=1/(1+@α3*power(@value-@αC,2))

set @sd=1/(1+@α4*power(@value-@αD,2))

set @se=0

end

else

begin

set @sa=1/(1+@α1*power(@value-@αA,2))

set @sb=1/(1+@α2*power(@value-@αB,2))

set @sc=1/(1+@α3*power(@value-@αC,2))

set @sd=1/(1+@α4*power(@value-@αD,2))

set @se=1/(1+@α5*power(@value-@αE,-2))

end

end

if @feature='hoping-target'

begin

set @x=abs(@value-@target)

if @x<=@αA

begin

set @sa=1; set @sb=0; set @sc=0;set @sd=0;set @se=0

end

else if @x<@αE

begin

set @sa=1/(1+@α1*power(@x-@αA,2))

set @sb=1/(1+@α2*power(@x-@αB,2))

set @sc=1/(1+@α3*power(@x-@αC,2))

set @sd=1/(1+@α4*power(@x-@αD,2))

set @se=0

end

else

begin

set @sa=1/(1+@α1*power(@x-@αA,2))

set @sb=1/(1+@α2*power(@x-@αB,2))

set @sc=1/(1+@α3*power(@x-@αC,2))

set @sd=1/(1+@α4*power(@x-@αD,2))

set @se=1/(1+@α5*power(@x-@αE,-2))

end

end

if @feature='hoping-large'

begin

if @value<=@αE

begin

set @sa=0;set @sb=0;set @sc=0;set @sd=0;set @se=1

end

else if @value<=@αA

begin

set @sa=0

set @sb=1/(1+@α2*power(@value-@αB,2))

set @sc=1/(1+@α3*power(@value-@αC,2))

set @sd=1/(1+@α4*power(@value-@αD,2))

set @se=1/(1+@α5*power(@value-@αE,2))

end

else

begin

set @sa=1/(1+@α1*power(@value-@αA,-2))

set @sb=1/(1+@α2*power(@value-@αB,2))

set @sc=1/(1+@α3*power(@value-@αC,2))

set @sd=1/(1+@α4*power(@value-@αD,2))

set @se=1/(1+@α5*power(@value-@αE,2))

end

end

if @feature='hoping-have'

begin

if @value=1

begin

set @sa=1;set @sb=0;set @sc=0;set @sd=0;set @se=0

end

else

begin

set @sa=0;set @sb=0;set @sc=0;set @sd=0;set @se=1

end

end

if @feature='hoping-no'

begin

if @value=1

begin

set @sa=0;set @sb=0;set @sc=0;set @sd=0;set @se=1

end

else

begin

set @sa=1;set @sb=0;set @sc=0;set @sd=0;set @se=0

end

end

--归一化处理

set @s=@sa+@sb+@sc+@sd+@se

set @sa=@sa/@s;set @sb=@sb/@s;set @sc=@sc/@s;set @sd=@sd/@s;set @se=@se/@s

end

else --没有评价数据

begin

set @sa=0;set @sb=0;set @sc=0;set @sd=0;set @se=0;set @score=0

end

set @score=5*@sa+4*@sb+3*@sc+2*@sd+1*@se –Calculate scoe

set @mld=dbo.maxlsd(@sa,@sb,@sc,@sd,@se) -- Calculate max membership by custom function “maxsld”

set @grade=''

if @mld>0

begin

if abs(@sa-@mld)<=0.001 set @grade=@grade+'A'

if abs(@sb-@mld)<=0.001 set @grade =@grade +'B'

if abs(@sc-@mld)<=0.001 set @grade =@grade+'C'

if abs(@sd-@mld)<=0.001 set @grade=@grade+'D'

if abs(@se-@mld)<=0.001 set @grade=@grade+'E'

end

insert into [Summary result of indicators]([Task No.], [Indicator No.], A, B, C, D, E, Grade, Score) values(@taskno,@bh,round(@sa,3),round(@sb,3),round(@sc,3),round(@sd,3),round(@se,3),@grade,round(@score,3))

fetch next from cursor1 into @bh, @feature,@target,@ αA,@ αB,@ αC,@ αD,@ αE,@α1, @α2, @α3, @α4, @α5

end

close cursor1

deallocate cursor1

--**Step 3: Get maximum level of non-final-level indicators and assign it to @level**

select @level=max(Level)-1 from [Evaluation task],Indicator where [Evaluation task].[Indicator system name]=Indicator. [Indicator system name] and [Task No.]=@taskno

--**Step 4: Determine membership of non-final-level indicators from low-level to high-level**

while @level>=1

begin

declare cursor1 cursor for select [Indicator No.] from [Evaluation task], Indicator where [Evaluation task].[Indicator system name]=Indicator. [Indicator system name] and [Task No.]=@taskno and Level=@level and [Final level]='N'

open cursor1

fetch next from cursor1 into @bh

while @@fetch_status=0 –For each non-final-level indicator of this level

begin

set @sa=0;set @sb=0;set @sc=0;set @sd=0;set @se=0;set @score=0

declare cursor2 cursor for select weight, A, B, C, D, E from Indicator, [Summary result of indicators] where [Summary result of indicators].[Indicator No.]=Indicator.[Indicator No.] and [Task No.]=@taskno and [Parent indicator No.]=@bh

open cursor2

fetch next from cursor2 into @weight, @A, @B, @C, @D, @E

while @@fetch_status=0

begin

set @sa=@sa+@weight*@A

set @sb=@sb+@weight*@B

set @sc=@sc+@weight*@C

set @sd=@sd+@weight*@D

set @se=@se+@weight*@E

fetch next from cursor2 into @weight, @A, @B, @C, @D, @E

end

close cursor2

deallocate cursor2

set @score=5*@sa+4*@sb+3*@sc+2*@sd+1*@se –Calculate score

set @mld=dbo.maxlsd(@sa,@sb,@sc,@sd,@se)

set @grade=''

if @mld>0

begin

if abs(@sa-@mld)<=0.001 set @grade=@grade+'A'

if abs(@sb-@mld)<=0.001 set @grade=@grade+'B'

if abs(@sc-@mld)<=0.001 set @grade=@grade+'C'

if abs(@sd-@mld)<=0.001 set @grade=@grade+'D'

if abs(@se-@mld)<=0.001 set @grade=@grade+'E'

end

insert into [Summary result of indicators]([Task No.],[Indicator No.], A, B, C, D, E, Grade, score) values(@taskno,@bh,round(@sa,3),round(@sb,3),round(@sc,3),round(@sd,3),round(@se,3),@grade,round(@score,3)) --添加新的隶属度

fetch next from cursor1 into @bh -- Deal with next non-final-indicator of this level

end

close cursor1

deallocate cursor1

set @level=@level-1 –Deal with higher level indicators

end

--**Step 5: Determine membership of evaluated mine**

set @sa=0;set @sb=0;set @sc=0;set @sd=0;set @se=0;set @score=0

declare cursor1 cursor for select weight, A, B, C, D, E from Indicator, [Summary result of indicators] where [Summary result of indicators].[Indicator No.]=Indicator.[Indicator No.] and [Task No.]=@taskno and [Parent indicator No.]=0

open cursor1

fetch next from cursor1 into @weight,@A,@B,@C,@D,@E

while @@fetch_status=0

begin

set @sa=@sa+@weight*@A

set @sb=@sb+@weight*@B

set @sc=@sc+@weight*@C

set @sd=@sd+@weight*@D

set @se=@se+@weight*@E

fetch next from cursor1 into @weight,@A,@B,@C,@D,@E

end

deallocate cursor1

set @score=5*@sa+4*@sb+3*@sc+2*@sd+1*@se – Calculate score

set @mld=dbo.maxlsd(@sa,@sb,@sc,@sd,@se)

set @grade=''

if @mld>0

begin

if abs(@sa-@mld)<=0.001 set @grade=@grade+'A'

if abs(@sb-@mld)<=0.001 set @grade=@grade+'B'

if abs(@sc-@mld)<=0.001 set @grade=@grade+'C'

if abs(@sd-@mld)<=0.001 set @grade=@grade+'D'

if abs(@se-@mld)<=0.001 set @grade=@grade+'E'

end

insert into [Overall evaluation result]([Task No.], A, B, C, D, E, Grade, Score) values(@taskno,round(@sa,3),round(@sb,3),round(@sc,3),round(@sd,3),round(@se,3),@grade,round(@score,3))

end
